# Involvement of calprotectin (S100A8/A9) in molecular pathways associated with HNSCC

**DOI:** 10.18632/oncotarget.7373

**Published:** 2016-02-13

**Authors:** Ali Khammanivong, Brent S. Sorenson, Karen F. Ross, Erin B. Dickerson, Rifat Hasina, Mark W. Lingen, Mark C. Herzberg

**Affiliations:** ^1^ Department of Diagnostic and Biological Sciences, University of Minnesota, Minneapolis, MN, USA; ^2^ Mucosal and Vaccine Research Center, Minneapolis VA Medical Center, Minneapolis, MN, USA; ^3^ Department of Pathology, University of Chicago, Chicago, IL, USA; ^4^ Department of Veterinary Clinical Sciences, University of Minnesota, St. Paul, MN, USA; ^5^ Masonic Cancer Center, University of Minnesota, Minneapolis, MN, USA

**Keywords:** calprotectin, S100A8/A9, cell cycle, differentiation, carcinogenesis

## Abstract

Calprotectin (S100A8/A9), a heterodimeric protein complex of calcium-binding proteins S100A8 and S100A9, plays key roles in cell cycle regulation and inflammation, with potential functions in squamous cell differentiation. While upregulated in many cancers, S100A8/A9 is downregulated in squamous cell carcinomas of the cervix, esophagus, and the head and neck (HNSCC). We previously reported that ectopic S100A8/A9 expression inhibits cell cycle progression in carcinoma cells. Here, we show that declining expression of *S100A8*/*A9* in patients with HNSCC is associated with increased DNA methylation, less differentiated tumors, and reduced overall survival. Upon ectopic over-expression of S100A8/A9, the cancer phenotype of S100A8/A9-negative carcinoma cells was suppressed in vitro and tumor growth in vivo was significantly decreased. MMP1, INHBA, FST, LAMC2, CCL3, SULF1, and SLC16A1 were significantly upregulated in HNSCC but were downregulated by S100A8/A9 expression. Our findings strongly suggest that downregulation of S100A8/A9 through epigenetic mechanisms may contribute to increased proliferation, malignant transformation, and disease progression in HNSCC.

## INTRODUCTION

Malignant transformation in squamous cell carcinomas (SCC) remains a perplexing process that results in abnormal cellular differentiation, invasivity, and growth regulation, all hallmarks of cancer. In carcinoma cells, calprotectin (S100A8/A9; MRP8/14), a heterodimeric complex of calcium-binding proteins, S100A8 and S100A9, appears to suppress cell cycle progression, growth, and migratory invasion by regulating cell differentiation and adhesion to extracellular matrix (ECM) [1-5]. Encoded by corresponding genes located in chromosomal locus 1q21.3 of the epidermal differentiation complex (EDC), S100A8/A9 is constitutively expressed in healthy squamous mucosal epithelial cells.

In different cancers, S100A8/A9 expression varies with cell and tissue of origin, but it is widely reported to be downregulated in cells from head and neck squamous cell carcinoma (HNSCC) [6-12]. The biological effects of S100A8/A9 depend on the extra- or intracellular localization of the protein complex. Likely released by infiltrating polymorphonuclear leukocytes, macrophages [13, 14], and by epithelial cells, extracellular S100A8/A9 in the tumor microenvironment is associated with inflammation-induced tumor progression [15] and may serve as a prognostic marker in some types of cancer [16, 17]. For example, S100A8/A9 is often abnormally elevated in tumors originating in tissues that are negative for the protein complex, whereas intracellular levels decrease in tumors such as HNSCC that originate from tissues with constitutive expression [1]. Intracellular S100A8/A9, therefore, may reflect the cancer phenotype.

In HNSCC, poor prognosis is highly associated with reduced S100A8/A9 protein levels [10]. Downregulation occurs at the transcriptional and protein expression levels, but no specific genetic mutations have been reported. Yet, S100A8/A9 appears to be an important regulatory protein affecting tumor behaviors. We have previously reported that expression of intracellular S100A8/A9 in carcinoma cells appears to activate protein phosphatase 2A (PP2A) and restore the cell cycle checkpoint at G2/M, suppressing growth and colony formation in soft agar [1]. S100A8/A9 may also affect squamous epithelial cell differentiation [18, 19]; loss of expression is associated with poorly differentiated tumors [19-21]. In contrast to myeloid-associated and extracellular functions of S100A8/A9, intracellular functions of S100A8/A9 in squamous epithelial cells may orchestrate gene networks critical in cellular development and tumorigenesis.

To understand S100A8/A9 in the larger context of gene networks, we analyzed the RNA sequencing (RNA-Seq) data from The Cancer Genome Atlas (TCGA) project (http://cancergenome.nih.gov) and other public datasets to decipher molecular and cellular functions associated with expression of S100A8/A9. The interrogation of the TCGA was based on a preliminary genome-wide differential expression profiles from transcriptomic analysis of microarrays developed from healthy human mucosal epithelium and primary HNSCC tissue samples obtained by laser capture microdissection (LCM). Genes regulated by HNSCC were subjected to correlation and pathway analyses to identify, for example, gene networks involved in growth and differentiation in association with S100A8/A9. In separate experiments, genes highly upregulated in HNSCC were further interrogated for their regulation by S100A8/A9 by ectopic overexpression in S100A8/A9-negative carcinoma cells and by knockdown of endogenous S100A8/A9 expression in HNSCC cells using short hairpin RNA (shRNA) interference. To validate that S100A8/A9 regulates gene networks affecting tumorigenesis, we determined whether loss of expression is associated with the development of HNSCC *in vivo*.

## RESULTS

### *S100A8* and *S100A9* gene expression and cellular differentiation-associated genes downregulated in human HNSCC and associated with poor survival

**Figure 1:**
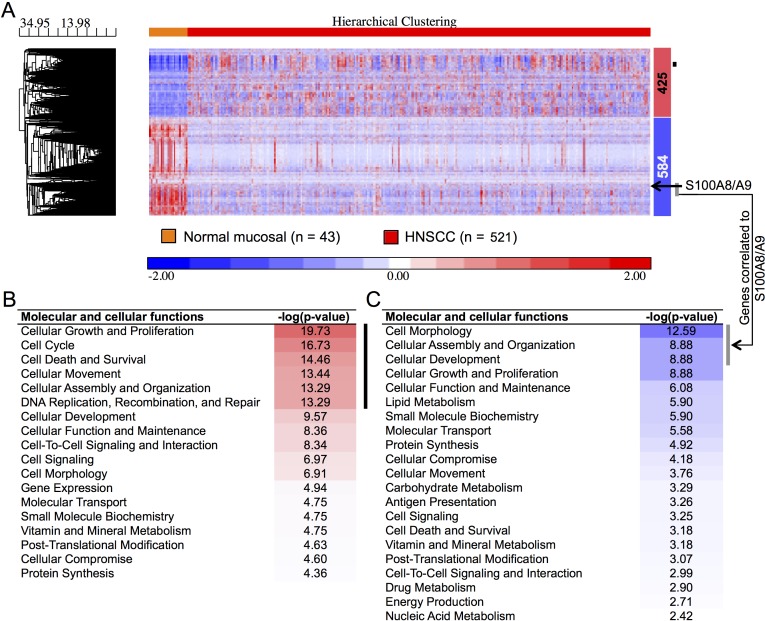
Differentially regulated genes and functions in HNSCC **A.** Two-dimensional hierarchical clustering heatmap of regulated genes showing clear separation of normal adjacent (*n* = 43) and HNSCC (*n* = 521) tissue samples from TCGA RNA-Seq data. Numbers of up- (red) and downregulated (blue) genes are shown to the right of the heatmap. The black arrow indicates expression profiles of S100A8/A9 in the cluster. The black vertical bar indicates genes negatively correlated to S100A9 (as a marker gene for S100A8/A9 protein complex) and gray vertical bar indicates a group of genes having strong positive correlation to S100A9 expression in HNSCC. Gene clustering dendogram is shown to left along with the distance (dissimilarity) between clusters on the horizontal axis. Molecular and cellular functions associated with differentially regulated genes are arranged with the most significantly enriched function on the top indicated with red shades for **B.** upregulated genes and blue shades for **C.** downregulated genes. Black and gray vertical bars indicate functions of genes negatively and positively correlated to S100A9, respectively, in HNSCC.

**Figure 2:**
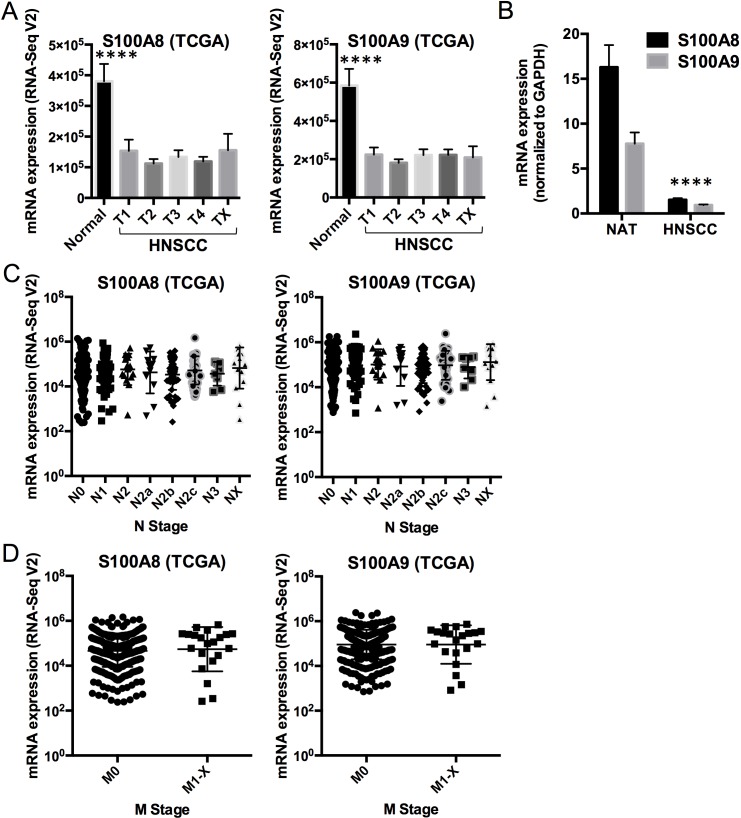
Comparison of S100A8 and S100A9 expression in normal mucosa and HNSCCs **A.** S100A8 and S100A9 calprotectin gene expression (mRNA) profiles in normal mucosal samples (*n* = 43) and HNSCC (*n* = 521, stratified by tumor grades 1 - 4, or T_X_ for unknown or no measurement; T_1_, *n* = 27; T_2_, *n* = 125; T_3_, *n* = 111; T_4_, *n* = 136; T_X_, *n* = 12) from TCGA RNA-Seq V2 data. Data shown as Mean ± SEM, with comparison between the normal and HNSCC samples averaged across all T grades (S100A8, −2.9-fold, ****p = 1.3×10^−17^; S100A9, −2.6-fold, ****p = 9.0×10^−15^). Statistical comparison was performed using two-tailed Student's *t*-Test with equal variance. **B.** Expression of S100A8 and S100A9 mRNA in normal adjacent tissues (NAT) and HNSCC normalized to GAPDH using qRT-PCR. Data shown as Mean ± SD (*n* = 3); ****p < 0.0001 (two-tailed Student's *t*-Test with equal variance) for both S100A8 and S100A9 in HNSCC compared to the NAT. **C.** S100A8 and S100A9 mRNA expression in HNSCC samples with different N grades from TCGA RNA-Seq V2 data. N_0_, *n* = 194; N_1_, *n* = 68; N_2_, *n* = 17; N_2a_, *n* = 12; N_2b_, *n* = 58; N_2c_, *n* = 38; N_3_, *n* = 8; N_X_, *n* = 16. Statistical comparisons were performed by ANOVA and no statistical difference was found among the sample groups. **D.** S100A8 and S100A9 mRNA expression in HNSCC with M = 0 (M_0_, *n* = 389) and M = 1 to X (M_1-X_, *n* = 21) from TCGA RNA-Seq V2 data. No statistical difference was found.

To understand potential regulatory roles of S100A8/A9, HNSCC and normal mucosal tissues were compared using the TCGA RNA-Seq provisional database. We found 5,525 genes that were differentially regulated in HNSCC compared to normal tissues (FDR < 0.05, fold ≥ 2). Of these, 425 upregulated and 584 downregulated genes were concordant with the gene sets identified previously with our older microarray data (Figure 1A). The large differences in the number of regulated genes identified initially by our microarray data and now by the RNA-Seq data from TCGA likely reflect variability in platforms, sample collection and processing, tumor sites, disease status, and sample sizes. We analyzed only differentially regulated genes from TCGA RNA-Seq data that were concordant with our microarray dataset. As predicted, pathway analysis showed that genes upregulated in HNSCC were associated with cellular growth and proliferation, cell cycle, cell death and survival, cellular movement, cellular assembly and organization, and DNA repair (Figure 1B). These upregulated cellular and molecular functions, included genes such as topoisomerase (DNA) II alpha 170kDa (TOP2A), fibronectin 1 (FN1), centromere protein F 350/400kDa (CENPF), and E2F transcription factor 7 (E2F7), were negatively correlated (*ρ* ≤ −0.30, *p* < 0.05, Spearman correlation) with *S100A8* and *S100A9* (as indicated by the black vertical bars, Figure 1A and 1B; Table S1). In contrast, genes downregulated in HNSCC were associated with cellular development and differentiation (Figure 1C) and showed strong positive correlations (*ρ* ≥ 0.30, *p* < 0.05, Spearman correlation) with *S100A8* and *S100A9* expression as indicated by the vertical gray bars (Figure 1A and 1C; Table S2). The gene descriptions and levels of correlation to S100A8/A9 are presented (Table S3). Expression of *S100A8* and *S100A9* was downregulated in HNSCC (*S100A8*, 2.9-fold, FDR = 1.1×10^−16^; *S100A9*, 2.6-fold, FDR = 6.8×10^−14^), but was not correlated with tumor size (T stage) (Figure 2A). Expression levels of both S100A8 and S100A9 in HNSCC compared to the normal adjacent tissue (NAT) was confirmed using quantitative real-time RT-PCR (qRT-PCR) (Figure 2B) and other published data through Oncomine^®^ Research Edition (www.oncomine.org) (data not shown). Downregulation of *S100A8* and *S100A9* genes in HNSCC did not correlate with regional lymph node involvement (N stage) (Figure 2C) or distant metastases (M stage) (Figure 2C and 2D), suggesting that S100A8/A9 dysregulation contributes to initiation of malignancy.

To show whether reduction of *S100A8* and/or *S100A9* expression contributes to HNSCC tumorigenesis, we enumerated the number of HNSCC samples (TCGA data) in which *S100A8*, *S100A9*, or both were downregulated relative to the mean expression in normal adjacent samples (Figure S1A). In TCGA dataset, *S100A8* and *S100A9* were both reduced in 471 (90.4%) out of 521 HNSCC samples, whereas *S100A8* or *S100A9* alone was only reduced in 11 (2.1%) and 8 (1.5%) samples, respectively. Based on TCGA RNA-Seq data, patients were then segregated by low (below average) or high (above average) expression of *S100A8/A9*. The median survival time for the low *S100A8* group (*n* = 275) was 35.5 months and for the high *S100A8* group (*n* = 132) was 64.8 months (*n* = 0.03) based upon Kaplan-Meier survival plots and log-rank (Mantel-Cox) tests (Figure S1B). The high S100A9 group (median survival = 56.4 months, *n* = 136) tended towards greater survival than the low S100A9 group (median survival = 35.9 months, *n* = 271), but the difference was not statistically significant *p* = 0.12) (Figure S1C).

### S100A9 downregulation in human HNSCC is highly correlated with DNA methylation

**Figure 3:**
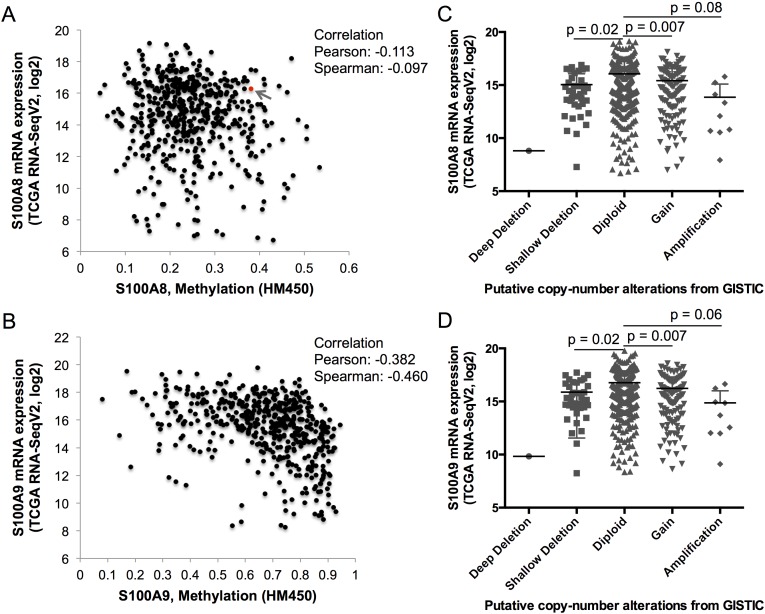
DNA methylation and copy number alteration in S100A8 and S100A9 TCGA RNA-Seq mRNA expression of S100A8 and S100A9, levels of genomic DNA methylation (shown as beta-values) determined from HumanMethylation450 (HM450) Illumina BeadArray, and putative copy number alterations from Genomic Identification of Significant Targets in Cancer (GISTIC) were analyzed using cBioPortal as described in the Methods. **A.** S100A8 and **B.** S100A9 mRNA expression levels and corresponding genomic DNA methylation. The red dot identified with a gray arrow represents a sample with K49R mutation in **A.** S100A8. Levels of **C.** S100A8 and **D.** S100A9 mRNA expression with respect to the level of putative copy number alterations. Deep deletion (putative homozygous deletion, *n* = 1), shallow deletion (putative heterozygous deletion, *n* = 37), diploid (unaltered, *n* = 332), gain (increase in one copy number, *n* = 114), and amplification (gain of more than one copy number, *n* = 9) are shown.

To identify potential mechanisms that might regulate *S100A8* and *S100A9* expression, TCGA RNA-Seq data was analyzed for DNA methylation and copy number alterations (or CNAs from Genomic Identification of Significant Targets in Cancer (GISTIC)). DNA methylation and *S100A8* expression did not correlate; one sample (sample ID# TCGA-CQ-5331) out of 528 cases analyzed showed a K49R missense mutation (Figure 3A, red dot with a gray arrow). Expression of *S100A9*, on the other hand, was negatively correlated with increasing levels of DNA methylation (Pearson and Spearman correlations ≈ −0.38 and −0.46, respectively) (Figure 3B), but no mutations were detected. Putative CNA was seen in one sample (ID# TCGA-D6-A6EO) with a deep deletion (homozygous deletion) of both *S100A8* and *S100A9*; shallow deletion (heterozygous deletion) of both genes resulted in significant reductions (p ≈ 0.02) in both *S100A8* and *S100A9* mRNA expression (Figure 3C and 3D). Interestingly, increased copy number in both *S100A8* and *S100A9* was associated with significantly reduced mRNA expression (*p* ≈ 0.007); overall mRNA expression tended to decrease in association with copy number amplification (gain in multiple copy numbers). When the methylated regions in the proximal promoter and gene body of *S100A8* and *S100A9* were compared, normal adjacent tissues showed greater overall DNA methylation than in HNSCC samples (Figure S2A, S2B). Promoter methylation was inversely (*p* < 0.05, Pearson correlation) but weakly correlated to the levels of *S100A8* mRNA expression (Figure S3A). Promoter methylation in the *S100A9* gene, on the other hand, was strongly and inversely correlated to the expression levels of *S100A9* mRNA (Figure S3B, black vertical bars).

### S100A8/A9 downregulates HNSCC associated genes

**Figure 4:**
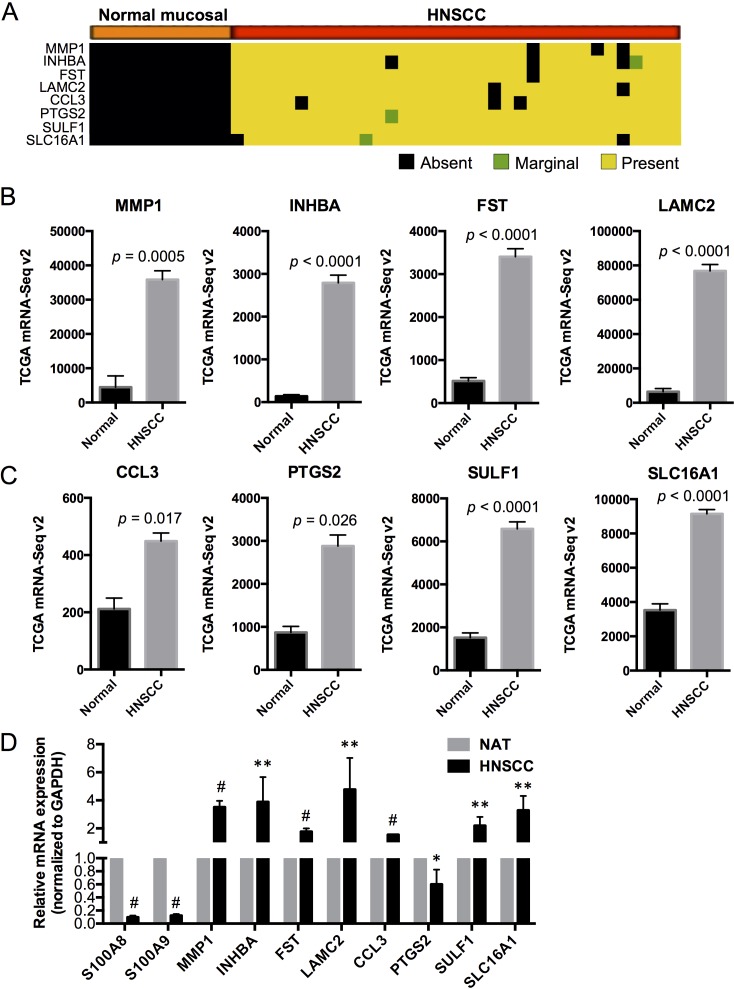
Expression profiles of putative HNSCC marker genes in normal and HNSCC samples in association with S100A8 and S100A9 **A.** Detection calls for each marker gene: Absent (no detection); Marginal (marginally expressed); Present (highly expressed). The genes were expressed and upregulated in > 90% of HNSCC samples but not detectable in any of the normal mucosal tissues. **B.**, **C.** Expression profiles of marker genes in normal adjacent and HNSCC tissues from TCGA RNA-Seq data. **D.** Relative mRNA expression of S100A8, S100A9 and marker genes in HNSCC compared to NAT, normalized to GAPDH using qRT-PCR. *p < 0.05, **p < 0.005, ^#^*p* < 0.0001. Relative qRT-PCR data are shown as Mean ± SD (*n* = 3 to 9).

**Figure 5:**
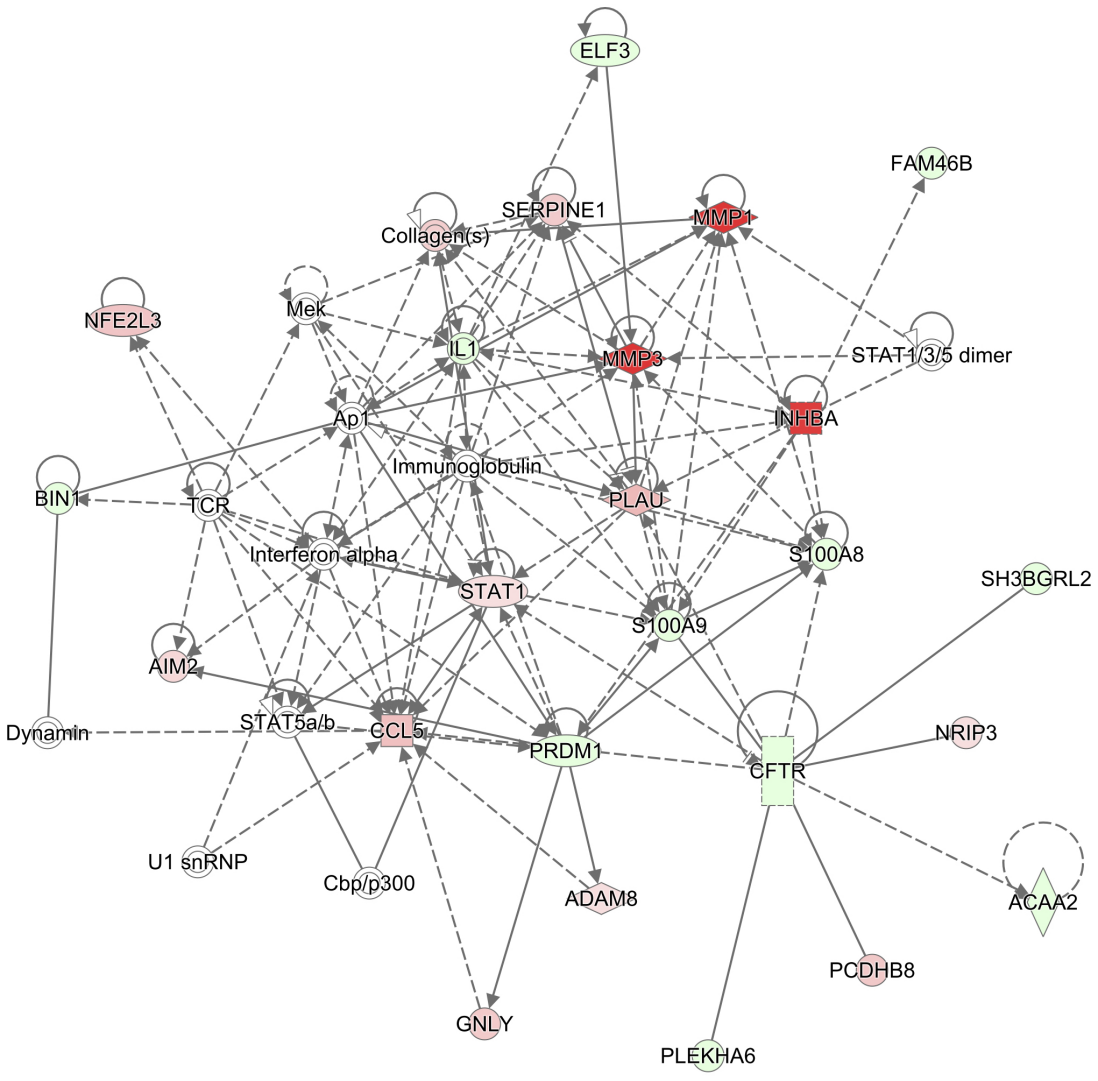
Network of S100A8/A9-associated genes in HNSCC Gene networks in HNSCC associated with *S100A8* and *S100A9* are identified using IPA. Genes regulated in HNSCC are compared with those in normal adjacent samples. HNSCC genes upregulated compared to normal are indicated in red symbols, downregulated in green, and unaffected genes are uncolored. In this analysis, genes associated with *S100A8* and *S100A9* are indicated by connections: dashed or solid lines. Solid and dashed lines with arrows represent direct and indirect (or predicted) relationships, respectively. Solid lines without arrows represent known protein-protein interactions. The S100A8/A9-associated genes in HNSCC are predicted to be involved in the inflammatory response, cardiovascular disease, and cellular movement.

Based on our preliminary microarray studies using differential analysis combined with expression detection calls, we found that matrix metallopeptidase 1 (MMP1), inhibin beta A (INHBA), follistatin (FST), laminin gamma 2 (LAMC2), chemokine (C-C motif) ligand 3 (CCL3), prostaglandin-endoperoxide synthase 2 (PTGS2), sulfatase 1 (SULF1), and solute carrier family 16 (or monocarboxylate transporter) member 1 (SLC16A1) were not detectable in any of the normal mucosal samples (with “Absent” detection calls) but were highly expressed (with “Present” detection calls in at least 90% of the HNSCC samples) and significantly upregulated in HNSCC (Figure 4A). These genes are highly associated with cellular proliferation, differentiation and carcinogenesis and encode proteins that localize to different cellular compartments (e.g., extracellular space, cytoplasm, plasma membrane and nucleus) [22, 23] and may serve as markers for HNSCC (summarized in Table S4). We now confirm that these genes are upregulated in HNSCC samples when compared to the normal adjacent tissues using TCGA RNA-Seq data (Figure 4B and 4C). The association between each putative marker gene and expression of S100A8 and S100A9 in HNSCC relative to normal adjacent tissues was validated by qRT-PCR (Figure 4D). In HNSCC, MMP1 and INHBA are among the highly upregulated genes in the network and show signaling relationships with *S100A8/A9* based on IPA gene network analysis (Figure 5).

**Figure 6:**
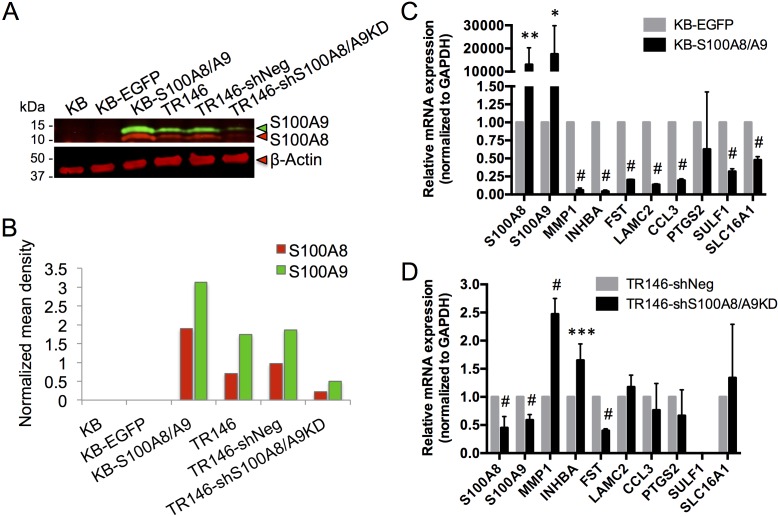
Expression of S100A8, S100A9 and putative marker genes in KB and TR146 cells **A.** Representative immunoblotting (from at least three independent repeats) of S100A8 (red) and S100A9 (green) in KB carcinoma cells with ectopic S100A8/A9 expression (KB-S100A8/A9) and control cells (KB parental and KB-EGFP empty vector control), and in TR146 cells with shRNA S100A8 and S100A9 knockdown (TR146-shS100A8/A9KD) and control cells (TR146 parental and TR146-shNeg scramble shRNA negative control). β-actin was used for total protein loading control. **B.** Densitometry analysis of the representative blot shown in A. Mean density of each band was background subtracted and the S100A8 and S100A9 signals were normalized to β-actin. **C.** Relative mRNA expression of S100A8, S100A9 and marker genes in KB-S100A8/A9 compared to KB-EGFP control, normalized to GAPDH as a reference gene by qRT-PCR. **D.** Relative mRNA expression of S100A8, S100A9 and marker genes in TR146-shS100A8/A9KD compared to TR146-shNeg control, normalized to GAPDH as a reference gene by qRT-PCR. *p < 0.05, **p < 0.005, ***p < 0.0005, ^#^*p* < 0.0001. Relative qRT-PCR data are shown as Mean ± SD (*n* = 3).

To show that loss of *S100A8/A9* expression contributes to HNSCC, we determined whether S100A8/A9 caused gain or loss of specific predicted functions using the S100A8/A9-negative carcinoma cell line, KB. In this line, we overexpressed S100A8/A9 ectopically [24] (Figure 6A and 6B). For comparison, S100A8/A9 was knocked down in the S100A8/A9-positive HNSCC cell line, TR146, using shRNA (Figure 6A and 6B). S100A8/A9 expression in KB cells (KB-S100A8/A9) significantly downregulated MMP1, INHBA, FST, LAMC2, CCL3, SULF1, and SLC16A1, but not PTGS2, when compared to KB cells expressing the empty vector control containing EGFP alone (KB-EGFP) (Figure 6C). Knockdown of S100A8/A9 in TR146 cells (TR146-shS100A8/A9KD) upregulated MMP1 and INHBA expression but unexpectedly reduced the expression level of FST (Figure 6D). LAMC2, CCL3, PTGS2, and SLC16A1 expression was unaffected by S100A8/A9 knockdown; SULF1 expression was undetectable. Glyceraldehyde-3-phosphate dehydrogenase (GAPDH), which was found to be unrelated to S100A8 and S100A9 expression based on transcriptomic analysis, was used as a reference gene for normalization in all qRT-PCR analyses.

### *S100A9* co-regulated with cellular differentiation genes in HNSCC; *S100A8/A9* downregulation associated with poor tumor differentiation

**Figure 7:**
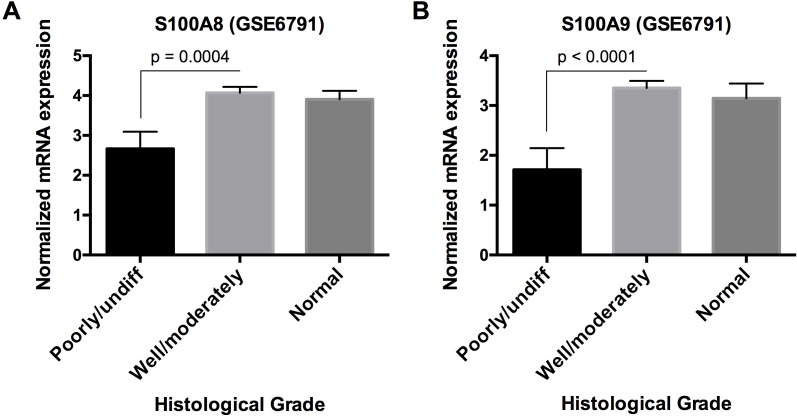
S100A8 and S100A9 mRNA expression in HNSCC with different histological grades Microarray data from GEO database, accession number GSE6791, was used to analyze **A.** S100A8 and **B.** S100A9 mRNA expression in poorly/undifferentiated (*n* = 10), well/moderately differentiated (*n* = 26), and normal samples (*n* = 14). Data are presented as Mean ± SEM. Statistical comparison was performed by two-tailed Student's *t*-test. No statistical significance was found between well/moderately differentiated and normal samples.

We then determined whether *S100A8/A9* expression and other downregulated genes were correlated. *S100A8* and *S100A9* were directly correlated (*ρ* ≥ 0.30, *p* < 0.05, Spearman correlation) with 107 (of 584) downregulated genes in HNSCC, which were enriched in functions including cellular development and differentiation, cell-to-cell signaling and interaction, and cell morphology as discussed above. Some genes showed known or predicted intergenic signaling relationships with *S100A8* and *S100A9*; these signaling relationships were associated with predicted functions in immunological cell development (Figure S4). Hence, *S100A8/A9* appears to be co-regulated within gene networks that regulate cellular differentiation, which may explain how loss of S100A8/A9 affects carcinogenesis. Indeed, *S100A8* and *S100A9* mRNA levels in poorly or undifferentiated HNSCC were significantly lower *p* = 0.0004 and *p* < 0.0001, respectively) than well or moderately differentiated tumors, which were indistinguishable from normal samples based on analysis of microarray data GSE6791 from the GEO database (Figure 7).

### S100A8/A9 affects KB cell morphology, actin organization and cellular adhesion

**Figure 8:**
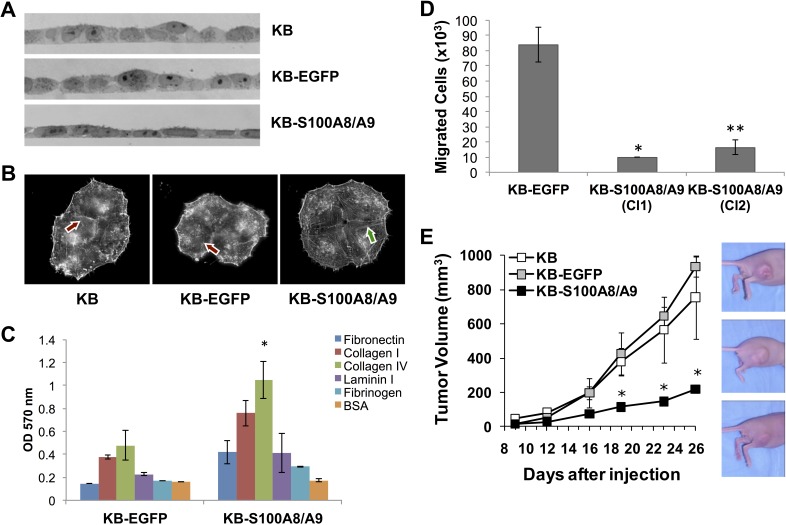
S100A8/A9 expression affects KB cell morphology, actin polymerization, cellular adhesion to ECM, cell migration *in vitro*, and tumor formation *in vivo* Images represent results from multiple experimental repeats. **A.** Bright-field images of cross sections of KB, KB-EGFP and KB-S100A8/A9 cell monolayers, showing a more flat and tightly adherent phenotype in S100A8/A9-expressing KB-S100A8/A9 cells. **B.** F-actin fluorescent microscopy displaying cells stained with Alexa Fluor 594-conjugated phalloidin. Extended and organized (long) actin filaments were more prominent in KB-S100A8/A9 (red arrow) than in KB and KB-EGFP cells (green arrow). **C.** KB-EGFP and KB-S100A8/A9 were tested for their adhesion to different ECM proteins coated on a tissue culture well. BSA coated well was used as a negative control. Data presented as Mean ± SD; *p < 0.05 (*n* = 2). **D.** Carcinoma cell extravasation and migration was performed in transwell chambers with membrane containing 8 μm pores. Two KB cell clones expressing S100A8/A9 (KB-S100A8/A9 Cl1 and Cl2) were compared with KB-EGFP control cells. Data presented as Mean ± SEM (*p < 0.05, *n* = 2; **p < 0.005, *n* = 6). **E.** Mice injected with KB-S100A8/A9, KB or KB-EGFP cells were compared for tumor burden. Data shown as Mean ± SEM (*p < 0.05) of two independent repeats; 5 mice per cell line were used in each repeat.

Since S100A8/A9 might be involved in regulation of cellular development and differentiation, morphology, and cytoskeletal organization (Table S2), we studied ectopic S100A8/A9 expression in KB carcinoma cells for changes in phenotype. When cultured on gelatin-coated glass cover slips, KB-S100A8/A9 cells appeared flatter and more adherent than the KB-EGFP and KB parental controls (Figure 8A). The adherent and spread morphology of KB-S100A8/A9 cells and S100A8/A9-positive TR146 cells appeared similar (data not shown). S100A8/A9-expressing KB cells also showed extended and organized F-actin microfilaments (Figure 8B, arrows), whereas KB and KB-EGFP cells showed punctate microfilament staining. To determine whether changes in morphology and cytoskeleton altered cellular adhesion, we tested KB cells for adhesion to different extracellular matrix (ECM) proteins. KB-S100A8/A9 cells showed greater adhesion to all ECM proteins than KB-EGFP control cells, adhering most effectively to collagen type IV (Figure 8C).

### S100A8/A9 suppressed cell migration and tumor growth

S100A8/A9 expression appeared to suppress cellular migration-associated genes MMP1 and LAMC2 and increase cellular adhesion to ECM proteins. To learn whether S100A8/A9 might affect cellular migration, we performed an *in vitro* tumor migration assay. Two S100A8/A9-expressing KB cell clones (KB-S100A8/A9 Cl1 and Cl2) showed significantly lower migration (*p* < 0.05) than KB-EGFP cells (Figure 8D), suggesting that S100A8/A9 may affect the migratory phenotype. Reduction of the migratory phenotype by forced expression of S100A8/A9 was consistent with diminished *in vitro* migration using a wound healing model and invasion in Matrigel coated transwell assays of KB-S100A8/A9 and TR146 HNSCC cells when compared to KB-EGFP and TR146-shS100A8/A9KD knockdown cells, as we recently reported [2].

To determine whether S100A8/A9 affects tumorigenicity, the KB cell lines were inoculated into the hind flanks of athymic nude mice and tumor formation was followed. When compared with KB-S100A8/A9 cells, KB and KB-EGFP cells formed significantly larger tumors over time (Figure 8E). Tumors produced by KB and KB-EGFP cells grew at similar rates from days 12 to 26, increasing in mean volume from 66 to 845 mm^3^. In contrast, the KB-S100A8/A9 cells formed small, slow growing tumors that increased in mean volume from 29 mm^3^ at day 12 to 220 mm^3^ at day 26.

## DISCUSSION

We now report for the first time that intracellular S100A8/A9 functions as a tumor suppressor *in vivo*. The genetic basis for the reduced tumorigenic activity was predicted from transcriptomic analysis and confirmed using *in vitro* and *in vivo* functional assays. We have previously reported that S100A8/A9 suppresses carcinoma growth *in vitro* by signaling cell cycle arrest at the G_2_/M checkpoint [1]. The tumor suppressor activity represents a novel function of intracellular S100A8/A9 in addition to conferring resistance to invasion of epithelial cells by invasive pathogens [24-27] and anti-inflammatory functions [14].

In the extracellular environment, S100A8/A9 is the key antimicrobial protein found in the complexes known as neutrophil extracellular traps (NETs) [28, 29]. S100A8/A9 is typically released by infiltrating leukocytes and macrophages, secretory cells, or damaged epithelial cells [13, 14] and, when free and uncomplexed, is considered an “alarmin,” signaling pro-inflammatory responses through toll-like receptor 4 (TLR4) and the receptor for advanced glycation end products (RAGE) [30-32]. In the tumor microenvironment, free extracellular S100A8/A9 is associated with tumor-associated inflammation and progression in certain types of cancer.

Depending upon cell lineages and tissue types, malignancies differentially regulate and express S100A8/A9. As we and others show, squamous epithelial cells lining the mucosa of the head and neck (e.g., oral cavity, nasopharyngeal, and oropharyngeal), esophagus, and cervix constitutively express *S100A8/A9* [6-8, 21, 33-35]. Carcinomas of these tissues show decreased S100A8/A9 protein complex and reduction in S100A8/A9 is correlated with poor tumor differentiation and increased carcinoma growth and invasiveness. In human primary tumors originating from tissues that normally express little or no S100A8 and S100A9 protein, such as skin, breast, thyroid, liver, gastric mucosa, prostate, ovary, bladder, and lung, S100A8/A9 levels are often elevated in association with tumorigenesis and cancer progression [1]. The basis for lineage-dependent expression of S100A8/A9 in health and cancer is unknown. In this report, however, we do explain how expression of *S100A8/A9* is likely controlled in HNSCC and how the cancer phenotype is affected.

To determine whether intracellular S100A8/A9 might regulate cell cycle progression and growth in primary human HNSCCs, we performed correlation analysis of the *S100A8* and *S100A9* mRNA expression profiles. Since the genes encoding S100A8 and S100A9 are located within chromosomal locus 1q21, which contains a cluster of (at least 43) genes known as the epidermal differentiation complex (EDC) [36], we predicted that S100A8/A9 induces cell cycle arrest and suppresses growth in HNSCCs by signaling cellular differentiation. If so, reduced levels of S100A8/A9 protein complex and resulting loss of function could promote loss of differentiation and growth control, contributing to carcinogenesis in HNSCC and resistance to chemoradiotherapy [37]. Using TCGA RNA-Seq data, we found that expression of both *S100A8* and *S100A9* was significantly downregulated in HNSCC. Reduction in levels of *S100A8-* and *S100A9*-specific mRNAs correlates with the decreases in S100A8/A9 protein levels reported in clinical specimens of HNSCC [7, 8, 33]. S100A8 and S100A9 protein levels also are lower in HNSCC than normal tissues using immunohistochemical (IHC) staining (The Human Protein Atlas, www.proteinatlas.org) and in independent studies using HNSCC tumor sections and tissue microarray IHC [9, 11]. In oropharyngeal squamous cell carcinoma, S100A12 in addition to S100A8 and S100A9 was downregulated as observed by IHC; simultaneous reduction in both S100A8 and S100A12 expression was associated with worse overall patient survival [38]. Whereas a small subset of TCGA HNSCC samples may show a slight reduction in mRNA expression, the overall expression of S100A12 in HNSCC tended to increase (non-significant) when compared to the normal adjacent tissue samples. S100A12 was not changed from normal in our HNSCC microarray data and in other transcriptomic datasets analyzed using Oncomine.org. We do show that S100A12 is weakly correlated to S100A8/S100A9 when compared to other gene transcripts. Since S100A12 did not appear to be broadly associated with HNSCC pathogenesis (TCGA data), we did not further analyze S100A12 although a complimentary role cannot be excluded.

Interestingly, *S100A8* and *S100A9* dysregulation was similar across all tumor grades (T) and was independent of nodal involvement (N) or distant metastasis (M). Although we did not compare *S100A8/A9* expression in subjects based on human papillomavirus (HPV) or smoking status, we recognize that HPV infection has been increasingly associated with tumorigenesis in HNSCC [39, 40], a risk factor independent of tobacco carcinogens and alcohol usage. Based on TCGA data (not shown), expression of *S100A8* and *S100A9* tended to decrease more in HPV-positive than in HPV-negative HNSCC but was not statistically different. In HNSCC, HPV infection can affect gene expression, signal transduction pathways, and tumorigenesis through epigenetic modifications [41, 42]. Since expression levels appeared unaffected by the tumor stage, suppression of *S100A8* and *S100A9* expression is likely an early event in the pathogenesis of HNSCC and is independent of HPV status and copy number gains. As disease progresses, S100A8/A9 levels appear unchanged. Whereas perhaps not contributing to reduced *S100A8* and *S100A9* expression in HNSCC relative to the normal adjacent tissues, promoter methylation is inversely correlated to the expression levels of *S100A8* and *S100A9* and appears associated with poor survival of HNSCC patients. Further studies will be needed to confirm this association.

The proliferative nature of HNSCC was reflected in the disproportionate upregulation of positive regulators of the cell cycle. Indeed, *TOP2A*, *FN1*, *CENPF*, and *E2F7* were inversely correlated with *S100A8* and *S100A9*. Whether these genes function upstream or downstream of S100A8/A9 is being addressed in a separate study.

Genes located within the EDC on chromosomal locus 1q21, including *S100A8* and *S100A9*, are essential to maintain normal epithelial phenotype; these genes are downregulated in HNSCC as we and others report [43, 44]. Loss of cellular differentiation or increased cellular dedifferentiation appears to be a key driver in malignant transformation of HNSCC and associated with poor prognosis and treatment outcomes [45-47]. As we show, S100A8/A9 and a significant number of gene products associated with cellular development and differentiation are downregulated in HNSCC. A subset of these downregulated genes show strong positive correlation with *S100A8* and *S100A9* expression and are enriched in functions associated with cellular development and differentiation, cell-to-cell signaling and interaction, cell morphology, and cellular assembly. *S100A8* and *S100A9* (and the S100A8/A9 protein complex) appear to be co-regulated and strongly suggested to function in these pathways. *S100A8* and *S100A9* expression levels show strong concordance across all HNSCC samples. Approximately 90% of the HNSCC samples analyzed show combined loss of S100A8 and S100A9. Only about 2% of the HNSCC samples show single loss of either S100A8 or S100A9 relative to the mean expression in normal samples. In TGCA, the data are insufficient to report HNSCC survival and patient tumor characteristics when only *S100A8* or *S100A9* was downregulated. Based on our current findings, the combined loss of *S100A8* and *S100A9*, and the resultant reduction in S100A8/A9 protein complex, is strongly suggested to be a key factor contributing to the pathogenesis of HNSCC. Reduced S100A8/A9 expression appears to promote the HNSCC phenotype, since loss of differentiation and cell-to-cell contact growth inhibition can dysregulate cell cycle progression and increase carcinoma growth.

Note that S100A8 and S100A9 proteins typically form heterodimeric multimers when both complex partners are present [48] in a manner requiring the structural integrity of the calcium-binding EF-hands but heterodimerization is independent of the availability of calcium [24]. The heterodimer is specifically recognized by the monoclonal antibody 27E10, which does not bind the individual subunits. Using 27E10, mucosal epithelial cells were found to express abundant S100A8/A9 in the cytoplasm [49]. Some HNSCC cells can express low levels of cytoplasmic S100A8/A9 [26, 27]. There are several lines of evidence that explain why singular expression of either S100A8 or S100A9 is uncommon. S100A8/A9 (calprotectin) null mice are S100A9^−/−^ [50, 51]; embryos lacking S100A8 fail early in development, and S100A9^−/−^ mice also fail to express S100A8. Hence, S100A9 alone within cells may be toxic. When we constructed S100A8/A9-positive transfectants in S100A8/A9-negative (no S100A8/A9 protein complex) human carcinoma cells, transfection of S100A9 without S100A8 was unsuccessful and cells died [24, 52]. Cells expressing only S100A8 survive but are not hardy. Hence, cell survival reflects selection for expression of the S100A8/A9 heterodimer, and *in vitro* studies of either S100A8 or S100A9 expression alone may not be experimentally feasible. Our observation that TGCA data rarely identified HNSCCs with only S100A8 or S100A9 being regulated is consistent with these findings.

The relationship between *S100A8/A9* and cell differentiation appears to manifest clinically. Poorly and/or undifferentiated HNSCC primary tumors expressed significantly lower *S100A8* and *S100A9* mRNA levels than the well and/or moderately differentiated tumors and normal samples. In fact, *S100A8* and *S100A9* expression levels were similar in normal specimens and well and/or moderately differentiated HNSCC. Thus, reduction in S100A8/A9 expression appears to characterize poorly and undifferentiated tumors associated with more aggressive disease, poor clinical prognosis, and treatment failures. Whether suppression of *S100A8* and *S100A9* expression is a driver or passenger, dysregulation during tumorigenesis cannot be ascertained from these analyses.

Candidate marker genes for HNSCC were identified. In HNSCC, MMP1, INHBA, FST, LAMC2, CCL3, SULF1, and SLC16A1 were upregulated using three independent modes of analysis: RNA-Seq data from TCGA, qRT-PCR, and microarrays. These genes were highly expressed and upregulated in HNSCC samples in TCGA and our study and expression was undetectable in normal mucosal tissues by microarray. MMP1, INHBA, and LAMC2 were reported previously as upregulated in HNSCC [53-55]. S100A8/A9 was confirmed to control expression of the candidate markers genes since MMP1, INHBA, FST, LAMC2, CCL3, SULF1, and SLC16A1 were all downregulated in KB cells (S100A8/A9-negative) with stable ectopic S100A8/A9 expression.

Note, however, that MMP1 expression was relatively low in KB cells, with qRT-PCR cycle threshold (CT) values in upper twenties (data not shown). We previously reported that S100A8/A9 suppressed expression and activity of MMP2 in KB cells while MMP1 mRNA and protein were nearly undetectable [2]. In S100A8/A9-expressing KB cells, MMP1 mRNA was slightly upregulated. The inconsistency in our findings may reflect low expression of MMP1 in KB cells, which may not serve to model MMP1 regulation in HNSCC. MMP2 expression, however, was constitutively high in normal tissues (data not shown), whereas MMP1 was undetectable. In HNSCC primary tumors, on the other hand, MMP1 was more highly expressed and upregulated than in normal tissues; MMP2 expression was not significantly upregulated in most HNSCCs.

MMP1 and INHBA expression appears to be regulated by S100A8/A9. In TR146 HNSCC cells, knockdown of S100A8/A9 increased expression of both MMP1 and INHBA, which are known to contribute to invasion and tumorigenesis, but other marker genes were unaffected. The discrepancy between TR146 cells and TCGA RNA-Seq data may reflect the heterogeneity in tumor behaviors across individuals and cell lines. Knockdown of S100A8/A9 tended to increase LAMC2 expression, which was implicated in invasion and recurrence of HNSCC [43]. Since shRNA reduced but did not abolish expression of S100A8/A9, we speculate that the HNSCC marker genes were only partially affected. We plan to confirm the S100A8/A9 regulation of LAMC2. Although SULF1 appears upregulated in gastric and other cancers [23, 56], this gene was not detectable in TR146 cells using qRT-PCR. Hence, SULF1 expression may reflect cell and tissue type specificity.

Lastly, the ability of carcinoma cells to de-differentiate into stem cell-like cells, migrate and invade surrounding tissues, metastasize to distant sites and resist apoptosis reflects malignant and metastatic potential [57, 58]. When compared to KB parental (S100A8/A9-negative) and KB-EGFP transfection control cells, KB cells expressing S100A8/A9 ectopically showed a more differentiated phenotype, with attenuated characteristics of carcinoma cells. For example, KB and KB-EGFP cells showed multiple F-actin puncta, suggesting increased assembly of focal adhesions and filopodia for migration and invasion [59-61] and higher levels of filamentous gamma 1 actin (ACTG1) expression (data not shown). KB cells lacking S100A8/A9 also showed decreased adhesion to the ECM proteins, and increased invasion and migration ability based on the presented and recently reported data using a wound healing model and Matrigel coated transwell assays [2]. Collectively, S100A8/A9 appears to inhibit the migratory phenotype and tumorigenic capability of KB carcinoma cells by restoring a more “benign” epithelial phenotype. Based on our *in vitro*, *ex vivo*, and *in silico* findings, we expected that the malignant and metastatic potential of carcinoma cells would be attenuated when S100A8/A9 is increased *in vivo*. Indeed, S100A8/A9-producing KB cells showed reduced tumorigenesis in nude mice in comparison to the parental and control KB-EGFP cells. S100A8/A9 expression was also found to suppress tumorigenesis when S100A8/A9-expressing TR146 cells and TR146-shS100A8/A9KD cells were injected in an orthotopic floor of mouth model in athymic nude mice (Sorenson et al. 2015, manuscript in preparation). Since TR146 and transfected KB-S100A8/A9 cells do not release detectable amounts of Mab27E10-reactive S100A8/A9 protein into the medium (data not shown), extracellular signaling through cell surface receptors such as TLR or RAGE by released S100A8/A9 as previously reported [30-32] is likely not associated with the functions described in this report. Collectively, our data strongly suggest that downregulation of intracellular S100A8/A9 may be a key mechanism responsible for an increase in proliferation and malignant transformation in HNSCC.

## MATERIALS AND METHODS

### RNA sequencing data processing from TCGA and statistical analysis

Level 3 RNA sequencing version 2 (RNA-Seq V2) dataset from 528 HNSCC cases that included 43 normal adjacent tissues (NAT) and 521 HNSCC samples was downloaded from The Cancer Genome Atlas (TCGA) website (http://cancergenome.nih.gov). Briefly, gene expression raw counts representing total transcripts mapped to the hg19 human reference genome using MapSplice alignment and quantitated by RSEM [62] were imported and processed using R data analysis software (www.r-project.org). Normalization and differential expression analysis was performed using edgeR Bioconductor package [63]. Statistical significance was calculated using false discovery rate (FDR) < 0.05 and fold-change ≥ 2.0 as cutoffs. Fold-change was determined as the ratio of means of HNSCC (*n* = 521) to normal (*n* = 43) samples. Fold down-regulation with ratio of means less than 1.0 is presented as −1/(ratio of means). Expression correlation analysis was performed using Spearman's rank method, unless stated otherwise, to account for non-linear relationships between gene products, with *ρ* (rho) ≤ −0.30 or ≥ 0.30 and *p* < 0.05 as statistical significance criteria.

### Copy number alteration and DNA methylation analysis

Copy number alternation (CNA) and DNA methylation analysis of TCGA HNSCC data was performed using the web-based cBioPortal [64, 65] TCGA data-mining interface (www.cbioportal.org). A total of 528 cases were analyzed for changes in copy number and DNA methylation. The methylated region in proximity and in the gene body of *S100A8* and *S100A9* was analyzed using Wanderer [66] and MEXPRESS [67].

### Tissue samples, laser-capture microdissection and RNA preparation for microarray

Oral mucosal epithelia from healthy volunteers and primary tumor tissues from HNSCC patients were harvested by laser-capture microdissection (LCM) and total RNA was extracted and amplified to generate mRNA for gene expression analysis. Primary HNSCC and normal oral mucosal tissue specimens were obtained from patients undergoing primary malignant resection surgery using a University of Chicago IRB-approved informed consent protocol. Tissues were obtained from 11 non-HNSCC subjects and 50 HNSCC patients with tumors in various TNM stages, ranging from T_1_ to T_4_ with N_0_ to N_3_, and M_B_ to M_C_. Fifty-four percent of the HNSCC patients were males and 38% females, with ages ranging from 26 to 90 years old (mean and median = 60). No demographic information was available for three patient specimens and ages were unavailable for three others. The tissue samples were handled under strictly maintained temperatures to prevent degradation of RNA. Immediately after collection, the samples were embedded in TissueTek OCT compound and snap frozen at −80°C. Frozen sections were cut to 5 - 8 μm thicknesses followed by a quick hematoxylin and eosin (H&E) staining protocol to identify and differentiate epithelial cells from stromal or infiltrating immune cells in the tissue sections. Briefly, sections were fixed in 70% ethanol for 30 s and stained with H&E followed by three dehydration steps of 30 s each in 70%, 95%, and 100% ethanol. Laser capture microdissection of epithelial cells was performed using a Leica AS LMD (Leica Microsystems, Wetzlar, Germany) immediately following the staining.

To extract RNA, the microdissected cells were directly placed in lysis buffer provided in the RNA purification kit (RNeasy Micro Kit; Qiagen, Valencia, CA) and homogenized before storing at −80°C. Total RNA was extracted from the captured cells with the RNeasy Micro Kit and treated with DNase to remove genomic DNA. The quantity and quality of purified RNA were determined immediately after purification with the Agilent 2100 BioAnalyzer (Agilent Technologies, Santa Clara, CA). The mRNA was labeled and hybridized to Affymetrix Human Genome U133 Plus 2.0 GeneChips.

### Target labeling and microarray hybridization and analysis

Target preparation was performed according to a standard protocol described in the Eukaryotic Target Preparation section of GeneChip^®^ Expression Analysis Technical Manual (Affymetrix Inc., Santa Clara, CA 95051). To generate biotin-labeled complementary RNA (cRNA) from the cDNA, double-stranded cDNA was synthesized from total RNA using a Two-Cycle Target Labeling protocol for HNSCC RNA samples or One-Cycle Target Labeling for KB cell RNA extracts, followed by an *in vitro* transcription (IVT) reaction. Biotin-labeled cRNA was then fragmented and hybridized to Affymetrix Human Genome U133 Plus 2.0 for HNSCC samples or U133A arrays for KB cell samples according to the manufacturer's protocol. The arrays were scanned and the images processed and analyzed using Affymetrix's GeneChip Operating Software (GCOS).

### Microarray expression data analysis

Data mining and statistical analyses were performed using Expressionist Analyst Pro (Genedata Inc., Waltham, MA) and Partek Genomics Suite (Partek Inc., St. Louis, MO). In brief, expression signals of all probes were first mapped to known gene symbols with median expression value of multiple probes for each gene using GENE-E (The Broad Institute of MIT and Harvard, http://www.broadinstitute.org/cancer/software/GENE-E), followed by Quantile normalization to ensure that gene expression values were comparable across multiple samples (arrays). In addition, gene probes were further filtered by detection calls. When comparing normal and HNSCC samples, HNSCC-specific putative marker genes were selected only when the gene detection call was “Present” and significantly upregulated in at least 90% of HNSCC samples but were “Absent” in all normal samples. To determine differential expression, ANOVA comparisons were performed in Partek Genomics Suite using a false discovery rate (FDR) < 0.05 as significance threshold and fold-change of 2.0-fold as cutoff. Fold-change was determined as the ratio of means of tumor to normal samples. Downregulation fold-change with ratio of means less than 1.0 is presented as −1/(ratio of means). Pathway analysis was performed using Ingenuity Pathways Analysis (IPA) (Ingenuity Systems, Inc., Redwood City, CA) to identify putative functions associated with regulated genes. These data are not MIAME-compliant since the original CEL data files are not available. By analyzing this database, we formulated questions, which were then used to interrogate TGCA. Comparisons of these primary tissue microarray gene expression data with TGCA showed great consistency as we report.

### Cell culture

KB-EGFP and KB-S100A8/A9 cells were maintained in modified Eagle medium (MEM) supplemented with 10% fetal bovine serum (FBS) and 700 μg of Geneticin (G418 sulfate) per mL in 5% CO_2_ at 37°C to maintain a stable expression of S100A8/A9. KB wild-type cells were cultured similarly but without Geneticin. TR146 cells were cultured in 5% CO_2_ at 37°C in 50% Dulbecco's Modified Eagle Medium and 50% HAM F-12 medium supplemented with 10% FBS and the TR146 with either shRNA knockdown (TR146-shS100A8/A9KD) or scrambled shRNA control (TR146-shNeg) were cultured with 250 μg/mL of Geneticin to maintain stable knockdown. Cells were plated at 5,000 cells/cm^2^ in T75 flasks and grown until the epithelial monolayer reached approximately 70% confluency, or 72 h. Cells were then harvested by trypsinization for mRNA and protein expression analysis.

### Epithelial cell lines with stable S100A8/A9 expression

The S100A8/A9-negative KB carcinoma cell line was transfected to express *S100A8* and *S100A9* genes along with enhanced green fluorescent protein (EGFP) and neomycin resistant genes for selection (KB-S100A8/A9 cells) and compared to KB transfection control cells containing only the selection genes (KB-EGFP cells) as reported previously [24, 25]. The TR146 human buccal cell carcinoma cell line was provided by Reuben Lotan, MD Anderson Cancer Hospital, Houston, TX. TR146 cells constitutively express S100A8/A9. For *in vitro* HNSCC loss of function studies, S100A8 and S100A9 were knocked down in TR146 cells using short hairpin RNA (shRNA)-induced gene silencing as previously described [26].

### RNA extraction and expression analysis by qRT-PCR

The cells were trypsinized, centrifuged and collected as a pellet, and washed once with Dulbecco's phosphate-buffered saline without calcium and magnesium (PBS). Total RNA was extracted and purified using the RNeasy Mini Kit (Qiagen, Valencia, CA USA). The final total RNA was eluted in 50 μL RNase-free water. Total RNA samples were quantitated by spectrophotometry for absorbance at 260 nm and adjusted to equal concentrations. Total RNA samples from HNSCC tumor tissue and normal adjacent tissue (NAT) were pooled from three different HNSCC patients as previously described [1]. Reverse transcription reactions were performed using the iScript cDNA Synthesis kit (Bio-Rad, Hercules, CA USA). Comparative quantitative real-time RT-PCR (qRT-PCR) was performed using primers designed to specifically detect and amplify genes of interest with SYBR^®^ Green QPCR Master Mix (Agilent Technologies Inc., Jolla, CA USA) and normalized to GAPDH as a reference gene.

### Cellular morphology and *in vitro* anchorage-independent growth

For microscopic examination, KB, KB-EGFP and KB-S100A8/A9 cells were grown under identical conditions on gelatin-coated glass cover slips. Cross-sectional bright field microscopic imaging was performed. Polymerization of actin was visualized by staining the cells with phalloidin conjugated to Alexaflor 594, followed by fluorescence microscopic imaging.

### Cellular adhesion to extracellular matrix

Cell adhesion assays were performed on extracellular matrix (ECM) protein coated wells using the CytoSelect^TM^ 48-Well Cell Adhesion Assay Kit based on the manufacturer's protocol (Cell Biolabs, Inc., San Diego, CA USA). At approximately 70% confluency (∼ 48 h in culture), cells were synchronized by overnight serum starvation. Serum starved cells were then harvested by trypsinization and resuspended in serum free media at 1 × 10^6^ cells/mL. Wells pre-coated with ECM (fibronectin, collagen I, collagen IV, laminin I, or fibrinogen) or BSA were seeded with 1.5 × 10^5^ of serum-starved cells per well, followed by 90 min incubation at 37°C in 5% CO_2_. BSA-coated wells served as a negative control. The growth medium from each well were then aspirated and each well was gently washed 4 times with 250 μL PBS. After the last wash, the adherent cells were stained with 200 μL of Cell Staining Solution (as provided in the assay kit) for 10 min at room temperature, washed 4 times with deionized water and air dried for 10 min. Stained cells were enumerated by addition of 200 μL per well of Extraction Solution (as provided in the assay kit) and incubated for 10 min on an orbital shaker at room temperature. Extracted samples (150 μL of each) were transferred to a 96-well microtiter plate and optical density (OD) was measured at 570 nm.

### Cell migration/invasion assay

Migration and invasion of carcinoma cells were tested using a transwell system (Costar 3428 with 8 μm pore size) (Thermo Fisher Scientific Inc., Waltham, MA USA). Cells growing in log phase (∼70% confluency; 48 h of growth) were synchronized by serum starvation overnight, followed by G_1_/S blockage with 2 μg/mL aphidicolin in MEM + 10% FBS for another 18 h. 1 × 10^6^ synchronized cells in serum-free medium were then seeded in a transwell chamber and allowed to settle for 30 min before MEM supplemented with 20% FBS was added to the outer well to stimulate migration and incubated at 37°C, 5% CO_2_ for 13 h. After 13 h of incubation, the media in both the transwell chamber and outer well were aspirated and the chamber and outer well were washed once with PBS. Migrated cells adhering to the bottom surface of the transwell membranes were harvested by trypsinization and counted using a hemocytometer.

### *In vivo* tumorigenesis

To determine whether S100A8/A9 expression affected tumor formation in KB carcinoma cells, BALB/c athymic nude mice (*n* = 5 per cell group per experiment) were inoculated subcutaneously with KB, KB-EGFP or KB-S100A8/A9 cells per hind flank at 1 × 10^6^ cells per injection in PBS. At weekly intervals up to 4 weeks post injection, the largest and smallest diameters of each tumor were measured with a caliper. Tumor volume (V) was then calculated from the equation, *V = π/6* × *larger diameter* × *(smaller diameter)^2^*. Tumor growth was determined as the change in tumor volume (V) over time. Experiments were performed in accordance to all federal guidelines and procedures were reviewed and approved by the University of Minnesota Institutional Animal Care and Use Committee.

## SUPPLEMENTARY MATERIAL FIGURES AND TABLES


