# Supplementary material for: Involvement of calprotectin (S100A8/A9) in molecular pathways associated with HNSCC
Source: Oncotarget. 2016 Feb 13;7(12):14029–47. doi: 10.18632/oncotarget.7373 (PMC4924696; doi:10.18632/oncotarget.7373)
Supplement: Supplementary file 1 [file oncotarget-07-14029-s001.pdf]

Involvement of calprotectin (S100A8/A9) in molecular pathways associated with HNSCC

Supplementary Material

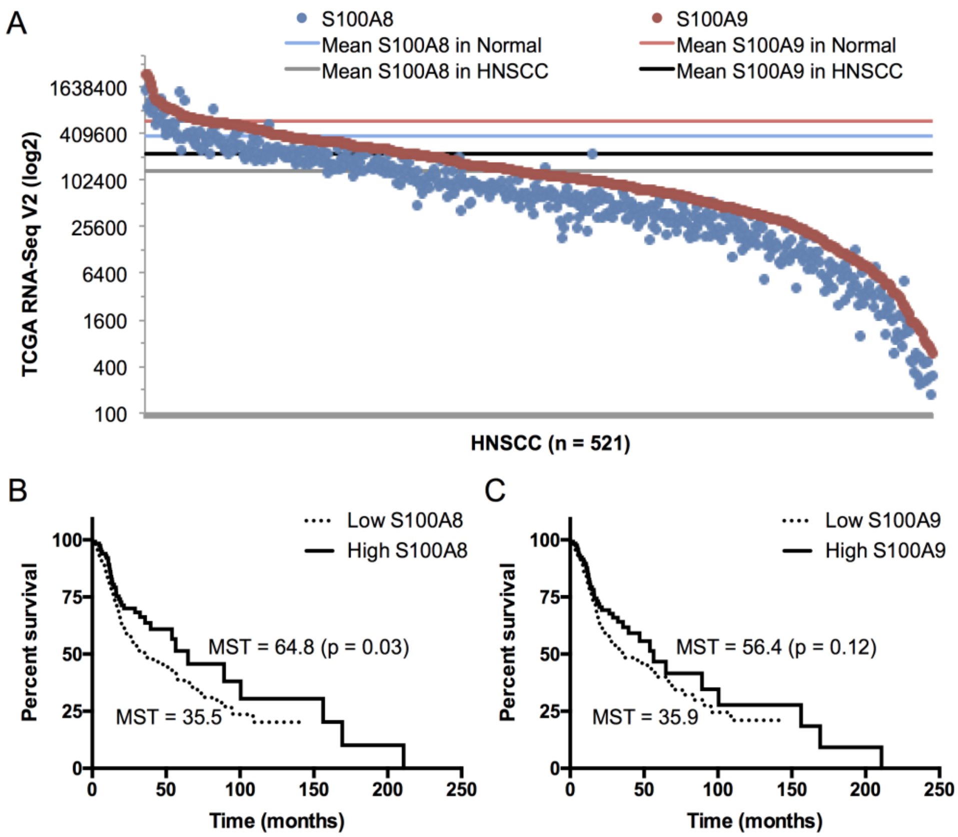

Figure S1. Patient survival with respect to *S100A8* and *S100A9* expression. Total cases from TCGA were separated into two groups and dichotomized into low and high *S100A8* and *S100A9* cohorts based on expression level below or above average in HNSCC. (**A**) Expression of *S100A8* and *S100A9* across all HNSCC samples and sorted based on *S100A9* mRNA level. Solid lines indicate mean expression of *S100A8* and *S100A9* in normal and HNSCC samples. (**B**) Kaplan-Meier survival plot of low (n = 275) and high (n = 132) S100A8 samples in HNSCC. (**C**) Kaplan-Meier survival plot of low (n = 271) and high (n = 136) S100A9 samples in HNSCC. Statistical analysis was performed using log-rank (Mantel-Cox) test. MST: Median survival time (months).

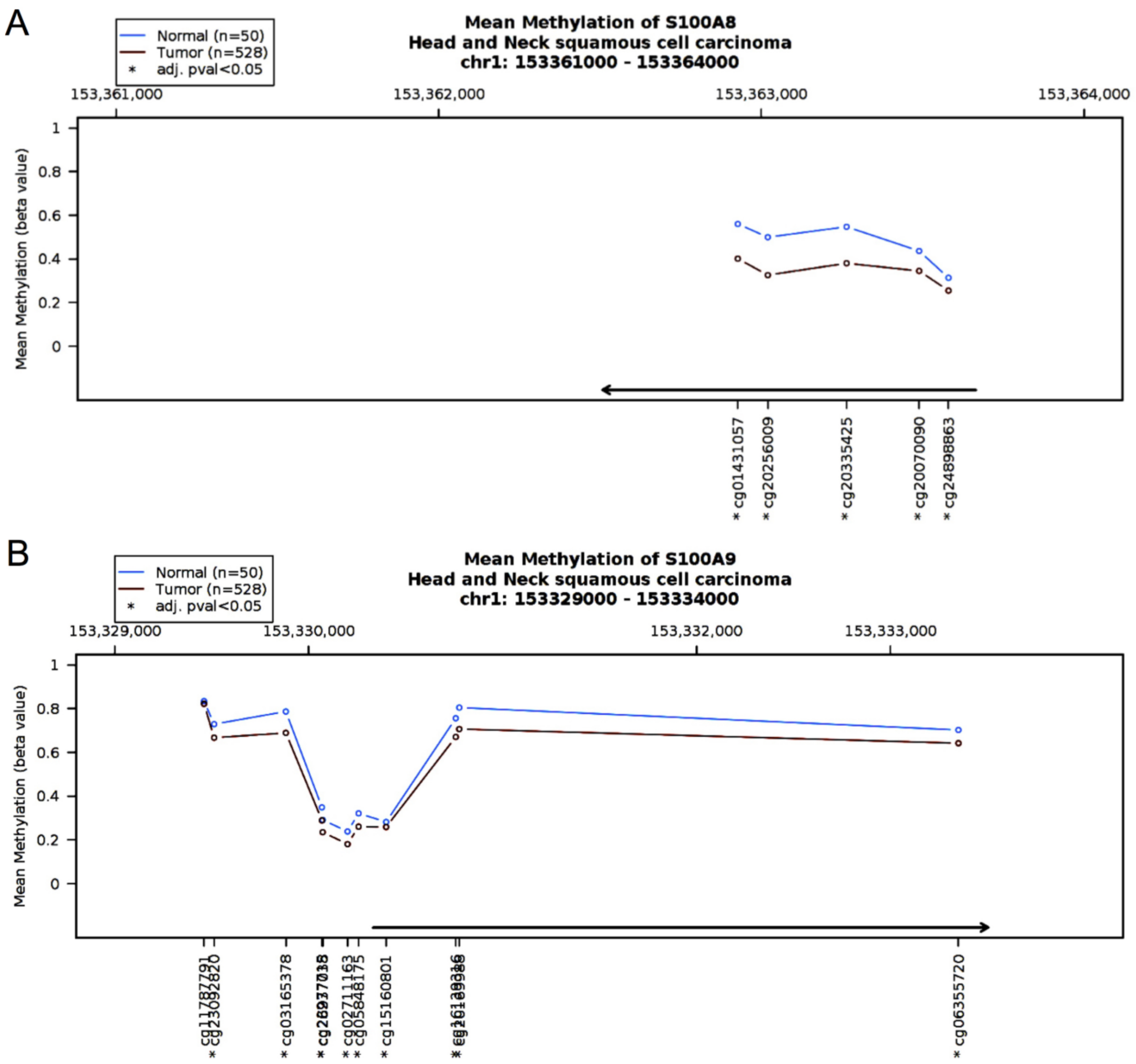

Figure S2. Methylation analysis of S100A8 and S100A9 genes in normal and HNSCC samples from TCGA data. (**A**) Mean methylation of S100A8 in normal and HNSCC tissues shown as beta values. Five different methylation probes are shown along the bottom axis. (**B**) Mean methylation of S100A9 in normal and HNSCC tissues shown as beta values. Eleven different methylation probes are shown along the bottom axis. Black arrow represents location and direction of the gene. Number on the top axis represents chromosomal base pair location.

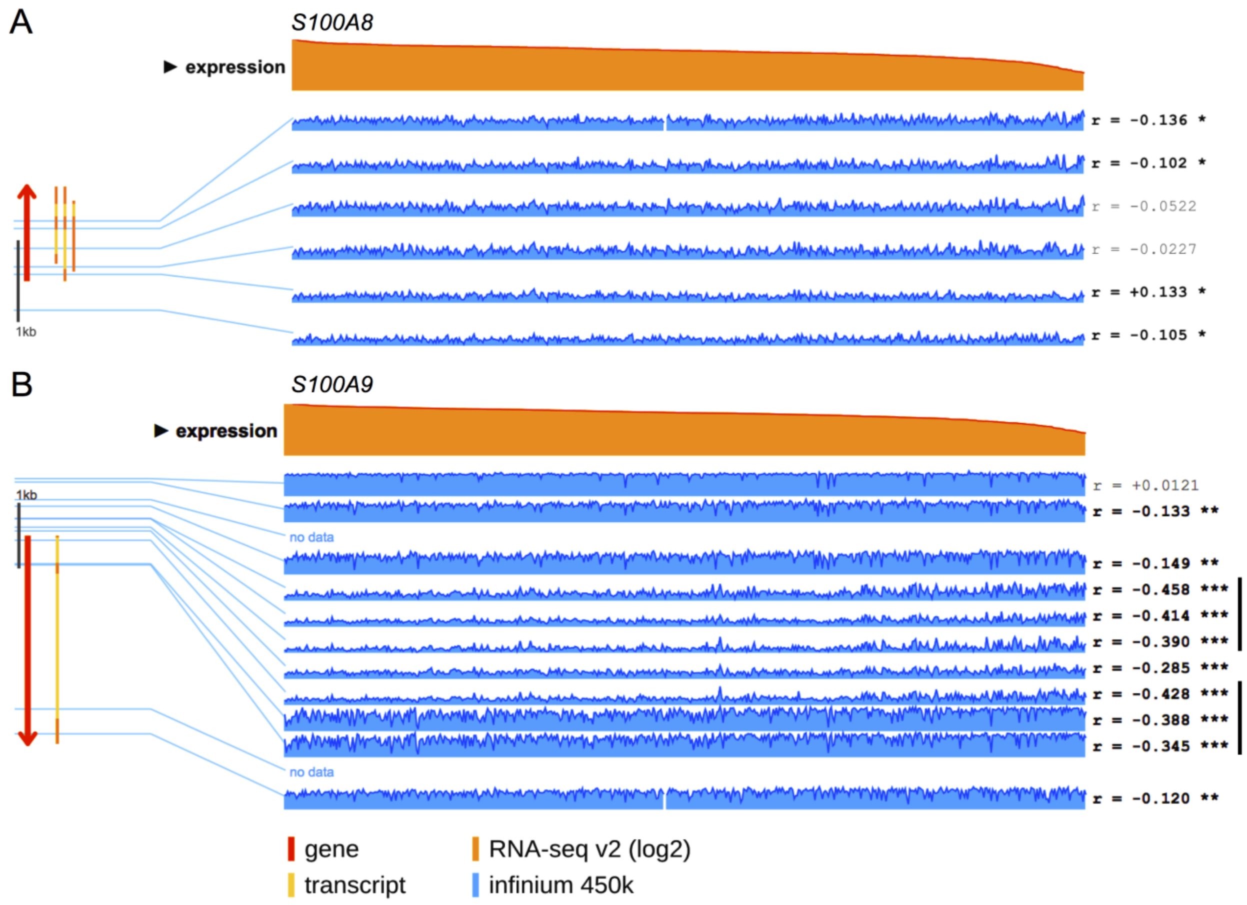

Figure S3. S100A8 and S100A9 mRNA expression with respect to different methylated promoter and gene regions from TCGA data. (**A**) Levels of methylation in the upstream promoter region and gene body, *S100A8* mRNA expression, and correlation between each methylation probe and *S100A8* expression. (**B**) Levels of methylation in the upstream promoter region and gene body, *S100A9* mRNA expression, and correlation between each methylation probe and *S100A9* expression. Pearson correlation coefficient (r) is shown to the right of each methylation probe with light-blue line to the left indicating chromosomal location relative to the gene (red arrow). X-axis represents samples and the height of each sample represents methylation beta value (blue) or mRNA expression (orange; TCGA RNA-Seq V2 data). *p < 0.05, **p < 0.01, ***p < 0.001 by Wilcoxon rank-sum test. Black bars indicate strong inverse correlation between methylated upstream promoter and N-terminus regions and mRNA expression of *S100A9*.

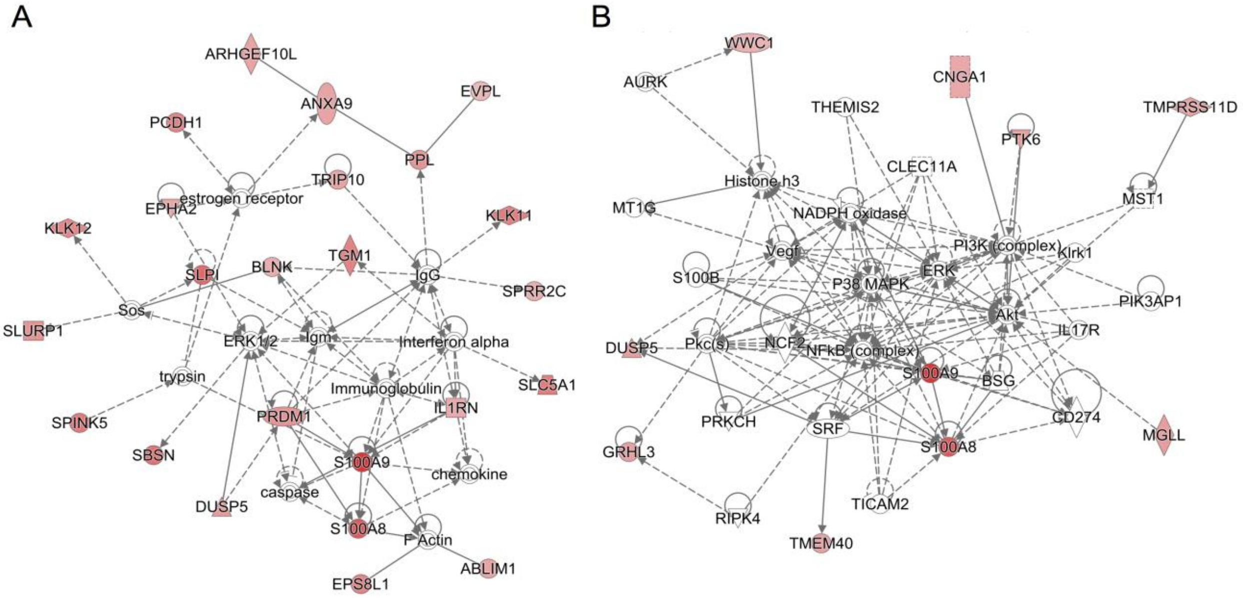

Figure S4. The network of *S100A9*-correlated and downregulated genes in HNSCC*.* When compared to genes expressed by normal mucosa, we performed a *t*-test to identify genes downregulated in HNSCC. Correlation analysis established the strength of correlation with *S100A9* (used as a surrogate gene for *S100A8/A9*). Genes correlated with *S100A9* are shown in red symbols, with a darker shade of red representing stronger correlation and a lighter shade of red representing lower correlation. Uncolored symbols indicate genes that were not significantly correlated with *S100A9* expression. Lines representing the intergenic relationships are explained in the legend of Figure 7. (**A**) Genes correlated with *S100A8/A9* were predicted using IPA to form a functional network based on known and predicted signaling pathways. These pathways are associated with dermatological diseases and conditions, hematological system development and function, and immune cell trafficking. (**B**) Network of genes related to *S100A8/A9* but only a few show correlations in HNSCC. Based on IPA, this gene network is predicted to participate in cellular function and maintenance, hematological system development and function, and the inflammatory response.

Table S1. Functions of genes negatively correlated to *S100A8* and *S100A9* and upregulated in HNSCC identified by TCGA RNA-Seq V2 data and confirmed with microarray. Functional analysis was performed using IPA, with the most significant ($p < 0.05$) molecular and cellular functions listed from the top.

Molecular and Cellular Functions	p-value	Molecules
Cell Morphology	2.47E-05-2.06E-02	COL10A1,CTHRC1,GAP43,SALL4,LAMC1,COL8A1,E2F7, HOXA10,FN1,CENPF,RAD54B,CDH11,HEY1
Cellular Growth and Proliferation	8.94E-05-1.9E-02	BCAT1,SPOCK1,TOP2A,CTHRC1,MSI1,GAP43,SALL4, LAMC1,COL8A1,P4HA1,MAGED4/MAGED4B,E2F7,HOXA10, FN1,SCN9A,COL11A1,CENPF,FNDC3B,FADS1,HEY1,CDH11
Cellular Development	1.69E-04-1.9E-02	TOP2A,CTHRC1,MSI1,SALL4,GAP43,LAMC1,E2F7,HOXA10, FN1,NID2,COL11A1,CENPF,FNDC3B,CDH11,HEY1
Cellular Assembly and Organization	5.27E-04-1.97E-02	TOP2A,FN1,NID2,CENPF,COL11A1,GAP43,LAMC1,P4HA1, CDH11
Cellular Function and Maintenance	5.27E-04-1.59E-02	FN1,SCN9A,COL11A1,CENPF,SALL4,GAP43,FNDC3B,HEY1, P4HA1
Cell Cycle	1.51E-03-1.97E-02	TOP2A,FN1,CENPF,E2F7
Cell Death and Survival	1.6E-03-1.43E-02	TOP2A,FN1,MSI1,E2F7
Cell-To-Cell Signaling and Interaction	1.6E-03-1.9E-02	TOP2A,FN1,SCN9A,NID2,LAMC1,CDH11
Cellular Compromise	1.6E-03-7.97E-03	TOP2A,FN1
Cellular Movement	1.6E-03-1.75E-02	TOP2A,FN1,GAP43,LAMC1,CDH11
DNA Replication, Recombination, and Repair	1.6E-03-1.97E-02	TOP2A,FN1,CENPF
Lipid Metabolism	1.6E-03-9.56E-03	FN1,FADS1
Molecular Transport	1.6E-03-9.56E-03	BCAT1,FN1
Protein Trafficking	1.6E-03-3.2E-03	FN1
Small Molecule Biochemistry	1.6E-03-1.43E-02	BCAT1,TOP2A,FN1,CHST7,FADS1,HEY1,P4HA1
Amino Acid Metabolism	3.2E-03-1.43E-02	BCAT1,HEY1,P4HA1
Carbohydrate Metabolism	3.2E-03-9.56E-03	FN1,CHST7
Drug Metabolism	3.2E-03-3.2E-03	TOP2A
Post-Translational Modification	4.79E-03-4.79E-03	P4HA1
Cellular Response to Therapeutics	7.97E-03-1.43E-02	FN1
Gene Expression	7.97E-03-1.77E-02	HOXA10,FN1,HEY1

Table S2. Functions of genes positively correlated to *S100A8* and *S100A9* and down-regulated in HNSCC identified by TCGA RNA-Seq V2 data and confirmed with microarray. Functional analysis was performed using IPA, with the most significant ($p < 0.05$) molecular and cellular functions listed from the top.

Molecular and Cellular Functions	p-value	Molecules
Cellular Development	7.85E-07-4.93E-02	SPINK5,FLG,PDCD4,TGM1,SCEL,TRIP10,EPHA2,PRDM1,SPINK7, WWC1,TGM3,NMU,IL1RN,TGM5,GRHL3,EVPL,CAST,BLNK
Cell-To-Cell Signaling and Interaction	3.07E-04-4.45E-02	PINK1,PTK6,SLPI,EPS8L2,PRDM1,EPHA2,WWC1,NMU,IL1RN, ECM1,CAST,FUT3,DUOX2,MGLL,MPP7
Free Radical Scavenging	6.65E-04-6.65E-04	PINK1,DUOXA2,DUOX2
Small Molecule Biochemistry	6.65E-04-4.93E-02	PINK1,SULT2B1,DHRS9,GDPD3,TGM1,SLPI,DUOXA2,NMU, SLURP1,IL1RN,CYP2C18,RDH12,GRHL3,SLC5A1,CES2,DUOX2, DGAT2,MGLL
Cell Morphology	6.91E-04-4.75E-02	SPINK5,PINK1,MAL,TRIP10,EPS8L2,EPHA2,PRDM1,WWC1,RHCG, IL1RN,ATP6V0A4,GRHL3,CAST,SCNN1B,BLNK,DGAT2,MPP7
Cellular Assembly and Organization	6.91E-04-4.93E-02	SPINK5,PINK1,MAL,TGM1,TRIP10,EPS8L2,EPHA2,GRHL3,CAST, EPS8L1,ZFP36,DGAT2,MPP7
Molecular Transport	6.91E-04-4.93E-02	RHCG,NMU,SULT2B1,SLURP1,IL1RN,GRHL3,SLC5A1,SCNN1B, DGAT2,MGLL,RHBG
Post-Translational Modification	2.53E-03-4.02E-02	SPINK5,KLK12,TMPRSS11D,TGM1,PRSS3,EVPL,CAST,TGM3
Cell Death and Survival	3.46E-03-5E-02	PINK1,PDCD4,PTK6,TGM1,SLPI,PRDM1,EPHA2,CLIC3,SPINK7, IL1RN,USP2,CAST,SERPINB2,BLNK
Amino Acid Metabolism	5.04E-03-2.5E-02	IL1RN,TGM1
Cellular Compromise	5.04E-03-3.48E-02	NMU,PINK1,PDCD4,IL1RN,TGM1,FUT3,EPHA2
Cellular Function and Maintenance	5.04E-03-4.93E-02	PINK1,MAL,TRIP10,SLPI,EPS8L2,PRDM1,EPHA2,RHCG,IL1RN, ATP6V0A4,CAST,SCNN1B,MPP7
Cellular Growth and Proliferation	5.04E-03-4.93E-02	NMU,IL1RN,TRIP10,CAST,PRDM1,EPHA2,WWC1,SPINK7,MGLL
Drug Metabolism	5.04E-03-4.93E-02	IL1RN,DHRS9,CYP2C18,CES2
Lipid Metabolism	5.04E-03-4.93E-02	NMU,SULT2B1,SLURP1,IL1RN,DHRS9,RDH12,CYP2C18,GRHL3, GDPD3,SLPI,DGAT2,MGLL
Cellular Movement	8.92E-03-3.96E-02	TRIP10,SLPI,DEFB104A/DEFB104B,EPHA2,PRDM1,NMU,IL1RN, PRSS3,S100A14,CAST,FUT3,SERPINB2,ABLIM1,MGLL
Cell Cycle	1.01E-02-3.96E-02	CAST,KRT13,BLNK
Cell Signaling	1.01E-02-4.93E-02	IL1RN,ECM1,EPS8L1,EPS8L2,DUOX2,MGLL
Vitamin and Mineral Metabolism	1.13E-02-4.93E-02	DHRS9,RDH12
Carbohydrate Metabolism	1.51E-02-3.96E-02	NMU,B3GNT8,IL1RN,SLC5A1,TGM1,GDPD3,SLPI,MGLL,DGAT2
Energy Production	1.51E-02-3.96E-02	DHRS9,IL1RN
Protein Degradation	1.51E-02-4.02E-02	SPINK5,KLK12,PINK1,TMPRSS11D,IL1RN,USP2,PRSS3,CAST
Protein Synthesis	1.51E-02-4.02E-02	SPINK5,KLK12,PINK1,TMPRSS11D,IL1RN,USP2,PRSS3,CAST
Nucleic Acid Metabolism	2.5E-02-4.93E-02	IL1RN
Protein Folding	2.5E-02-2.5E-02	TGM1

Table S3. Genes correlated to *S100A8* and *S100A9* that were regulated in HNSCC as identified by TCGA RNA-Seq V2 data and confirmed with microarray data. Upregulated genes are shown with fold-changes shaded in red and downregulated genes with fold-changes shaded in green. Level of correlation of each gene to *S100A8* or *S100A9* are shown with Spearman's rank correlation coefficient (*ρ*; rho) and corresponding p-value.

		TCGA RNA-Seq V2			Affymetrix HG U133 Plus 2.0 Array		Correlation to S100A8 (TCGA RNA-Seq V2)		Correlation to S100A9 (TCGA RNA-Seq V2)	
Gene	Entrez gene description	FoldChange	p-value	FDR	FoldChange	p-value	ρ	p-value	ρ	p-value
S100A8	S100 calcium binding protein A8	-2.9	1.30E-17	1.13E-16	-1.2	0.075658	1.0	-	1.0	0.0
S100A9	S100 calcium binding protein A9	-2.6	9.04E-15	6.78E-14	-2.7	9.98E-10	1.0	0.0	1.0	-
BCAT1	branched chain amino-acid transaminase 1, cytosolic	3.2	4.20E-12	7.24E-12	5.0	0.005701	-0.32	4.13E-14	-0.31	8.85E-13
CDH11	cadherin 11, type 2, OB-cadherin (osteoblast)	4.1	4.14E-16	9.00E-16	2.9	0.0001983	-0.41	2.23E-22	-0.39	3.79E-20
CENPF	centromere protein F, 350/400kDa	2.7	2.68E-09	3.79E-09	2.2	0.000709	-0.33	4.62E-15	-0.34	1.31E-15
CHST7	carbohydrate (N-acetylglucosamine 6-O) sulfotransferase 7	3.6	8.45E-14	1.62E-13	4.8	0.006666	-0.39	1.66E-20	-0.40	1.33E-21
CNIH3	cornichon family AMPA receptor auxiliary protein 3	4.0	1.29E-15	2.73E-15	2.1	1.04E-08	-0.40	1.35E-21	-0.41	2.79E-22
COL10A1	collagen, type X, alpha 1	41.4	6.69E-64	4.57E-63	5.3	0.001977	-0.39	1.92E-20	-0.38	7.18E-19
COL11A1	collagen, type XI, alpha 1	23.3	3.80E-51	2.17E-50	7.8	0.01045	-0.32	7.37E-14	-0.30	2.40E-12
COL8A1	collagen, type VIII, alpha 1	4.6	4.10E-18	9.86E-18	8.4	0.003077	-0.41	1.53E-22	-0.38	6.00E-19
CTHRC1	collagen triple helix repeat containing 1	6.2	1.06E-23	3.16E-23	8.3	0.0001378	-0.39	1.71E-20	-0.39	8.51E-21
E2F7	E2F transcription factor 7	4.1	5.50E-16	1.19E-15	3.0	0.000949	-0.31	1.97E-13	-0.32	6.49E-14
FADS1	fatty acid desaturase 1	2.2	1.19E-06	1.37E-06	2.9	0.002679	-0.46	3.61E-29	-0.46	3.54E-29
FN1	fibronectin 1	7.4	4.59E-27	1.54E-26	7.1	0.009449	-0.40	6.61E-22	-0.39	3.46E-20
FNDC3B	fibronectin type III domain containing 3B	2.3	2.64E-07	3.18E-07	3.6	4.13E-11	-0.34	5.61E-16	-0.32	6.76E-14
GAP43	growth associated protein 43	2.2	7.80E-07	9.11E-07	2.6	1.68E-05	-0.32	7.82E-14	-0.31	7.04E-13
HEY1	hes-related family bHLH transcription factor with YRPW motif 1	2.6	1.11E-08	1.49E-08	3.9	0.003544	-0.52	6.14E-37	-0.52	5.46E-38
HOXA10	homeobox A10	11.8	1.20E-36	5.16E-36	5.5	2.23E-06	-0.34	1.34E-15	-0.33	1.10E-14
KDELC1	KDEL (Lys-Asp-Glu-Leu) containing 1	2.9	1.53E-10	2.35E-10	3.2	0.0001219	-0.30	1.26E-12	-0.30	1.28E-12
KLHL5	kelch-like family member 5	2.3	4.46E-07	5.30E-07	5.3	8.40E-08	-0.34	3.68E-15	-0.33	1.84E-14
KNTC1	kinetochore associated 1	2.7	2.68E-09	3.79E-09	2.9	5.16E-06	-0.30	1.63E-12	-0.32	1.02E-13
LAMC1	laminin, gamma 1 (formerly LAMB2)	2.1	3.15E-06	3.53E-06	3.1	0.0001804	-0.36	9.74E-18	-0.35	4.75E-16
MAGED4	melanoma antigen family D4B	3.6	7.72E-14	1.48E-13	3.8	0.006856	-0.34	3.84E-15	-0.35	2.22E-16
MSI1	musashi RNA-binding protein 1	4.7	2.29E-18	5.57E-18	2.5	0.000276	-0.41	3.50E-22	-0.40	3.51E-21
NID2	nidogen 2 (osteonidogen)	4.2	1.09E-16	2.44E-16	2.3	0.0122	-0.35	4.00E-16	-0.32	4.01E-14
NUDT11	nudix (nucleoside diphosphate linked moiety X)-type motif 11	4.3	8.13E-17	1.83E-16	5.3	0.00239	-0.41	4.47E-23	-0.42	1.62E-23
P4HA1	prolyl 4-hydroxylase, alpha polypeptide I	2.8	5.56E-10	8.20E-10	2.5	0.0008094	-0.41	4.72E-22	-0.39	1.29E-20
RAD51AP1	RAD51 associated protein 1	3.3	1.82E-12	3.21E-12	6.5	0.0001291	-0.35	1.69E-16	-0.37	1.88E-18
RAD54B	RAD54 homolog B (S. cerevisiae)	2.8	3.77E-10	5.65E-10	3.5	5.01E-05	-0.33	1.90E-14	-0.33	7.82E-15
RAI14	retinoic acid induced 14	2.2	1.27E-06	1.47E-06	2.1	0.01798	-0.36	4.68E-17	-0.33	1.96E-14
SALL4	spalt-like transcription factor 4	6.4	2.18E-23	6.50E-23	2.3	1.28E-06	-0.37	3.68E-18	-0.37	2.23E-18
SC65	synaptonemal complex protein SC65	3.4	6.35E-13	1.15E-12	3.5	0.000463	-0.33	4.06E-15	-0.34	1.29E-15
SCN9A	sodium channel, voltage gated, type IX alpha subunit	5.4	4.74E-21	1.28E-20	3.0	0.01365	-0.38	1.04E-19	-0.37	3.22E-18
SOX2OT	SOX2 overlapping transcript	3.4	8.93E-13	1.61E-12	9.3	0.01315	-0.33	6.62E-15	-0.34	1.45E-15
SPOCK1	sparc/osteonectin, cwcv and kazal-like domains proteoglycan (testican) 1	2.6	6.09E-09	8.33E-09	3.6	0.006622	-0.31	5.93E-13	-0.31	1.03E-12
TOP2A	topoisomerase (DNA) II alpha	2.7	2.23E-09	3.16E-09	6.3	1.20E-06	-0.36	2.42E-17	-0.35	1.68E-16
TRIM59	tripartite motif containing 59	2.3	3.26E-07	3.91E-07	2.1	0.004173	-0.38	4.05E-19	-0.38	5.14E-19
A2ML1	alpha-2-macroglobulin-like 1	-4.7	6.98E-42	3.33E-41	-5.7	8.95E-20	0.57	1.43E-46	0.58	2.31E-48
ABLIM1	actin binding LIM protein 1	-2.4	5.82E-12	9.93E-12	-2.5	1.30E-10	0.44	1.27E-26	0.46	1.61E-28
AIM1L	absent in melanoma 1-like	-2.3	6.16E-11	9.78E-11	-5.0	1.39E-15	0.73	1.28E-87	0.73	6.83E-88
ANKRD35	ankyrin repeat domain 35	-3.7	1.81E-28	6.33E-28	-6.5	5.52E-13	0.46	3.37E-28	0.43	3.41E-25
ANXA9	annexin A9	-6.0	1.24E-57	7.73E-57	-5.6	3.23E-05	0.38	4.12E-19	0.38	1.74E-19
ARHGEF10L	Rho guanine nucleotide exchange factor (GEF) 10-like	-2.9	2.71E-18	6.58E-18	-3.7	1.22E-11	0.53	1.44E-38	0.54	4.67E-41
ARSF	arylsulfatase F	-15.4	7.05E-156	1.19E-154	-2.1	3.84E-05	0.40	4.17E-21	0.40	1.92E-21
ATP6V0A4	ATPase, H+ transporting, lysosomal V0 subunit a4	-16.3	1.59E-170	2.96E-169	-7.2	4.87E-11	0.32	1.40E-13	0.32	4.27E-14
B3GNT8	UDP-GlcNAc:betaGal beta-1,3-N-acetylglucosaminyltransferase 8	-2.1	1.20E-08	1.61E-08	-3.8	3.12E-09	0.62	9.51E-57	0.64	1.03E-60
BLNK	B-cell linker	-2.9	1.09E-17	2.57E-17	-3.4	5.38E-15	0.51	2.70E-36	0.50	1.49E-34
BNIPL	BCL2/adenovirus E1B 19kD interacting protein like	-3.2	6.41E-22	1.81E-21	-4.7	5.85E-19	0.62	1.23E-56	0.61	2.37E-54
BSPRY	B-box and SPRY domain containing	-3.1	1.15E-20	3.07E-20	-2.7	1.24E-07	0.47	1.44E-30	0.46	1.32E-28
CAST	calpastatin	-2.0	6.91E-08	8.68E-08	-2.2	2.78E-11	0.37	6.78E-18	0.39	1.29E-20
CEACAM7	carcinoembryonic antigen-related cell adhesion molecule 7	-9.3	1.77E-98	1.82E-97	-3.2	0.007345	0.38	5.24E-19	0.39	6.68E-20
CES2	carboxylesterase 2	-2.3	1.55E-10	2.39E-10	-3.6	8.85E-18	0.39	2.79E-20	0.39	4.03E-20
CLCA4	chloride channel accessory 4	-14.7	2.32E-156	3.94E-155	-4.1	8.54E-07	0.47	2.63E-30	0.50	1.81E-34
CLDN17	claudin 17	-6.0	4.01E-58	2.53E-57	-3.1	0.0002058	0.56	2.37E-44	0.56	6.16E-45
CLIC3	chloride intracellular channel 3	-3.3	5.14E-23	1.51E-22	-7.5	4.64E-14	0.70	1.14E-77	0.70	2.80E-79
CNFN	cornifelin	-3.5	2.35E-25	7.48E-25	-2.7	1.76E-07	0.78	5.50E-107	0.77	2.60E-105
CNGA1	cyclic nucleotide gated channel alpha 1	-2.2	1.70E-09	2.43E-09	-2.2	8.53E-05	0.42	6.89E-24	0.43	2.81E-25
CRISP3	cysteine-rich secretory protein 3	-75.6	0	0	-84.1	3.44E-09	0.37	6.55E-18	0.38	8.85E-20
CRNN	cornulin	-27.7	7.09E-261	2.32E-259	-16.0	1.35E-23	0.50	4.20E-34	0.51	1.71E-35
CYP2C18	cytochrome P450, family 2, subfamily C, polypeptide 18	-3.7	5.07E-28	1.74E-27	-3.0	4.12E-08	0.44	1.39E-25	0.43	1.04E-24
DEFB104A	defensin, beta 104A	-2.4	1.26E-05	1.35E-05	-2.1	0.0006802	0.32	8.04E-14	0.32	9.25E-14
DGAT2	diacylglycerol O-acyltransferase 2	-4.0	1.13E-32	4.40E-32	-2.2	0.0001155	0.44	3.46E-26	0.43	3.87E-25
DHRS9	dehydrogenase/reductase (SDR family) member 9	-3.3	3.31E-23	9.80E-23	-2.8	7.15E-06	0.51	2.18E-36	0.54	4.95E-41
DNASE1L3	deoxyribonuclease I-like 3	-5.7	7.59E-55	4.54E-54	-3.5	3.73E-09	0.34	1.39E-15	0.34	3.85E-15
DUOX2	dual oxidase 2	-2.1	2.10E-08	2.76E-08	-2.3	3.78E-07	0.31	5.26E-13	0.34	3.15E-15
DUOXA2	dual oxidase maturation factor 2	-2.3	4.17E-11	6.69E-11	-2.0	0.01596	0.59	8.28E-51	0.61	6.25E-54
ECM1	extracellular matrix protein 1	-3.8	1.58E-29	5.65E-29	-6.5	2.12E-15	0.48	7.85E-31	0.49	2.73E-33
EPHA2	EPH receptor A2	-2.0	6.17E-08	7.79E-08	-2.3	6.68E-11	0.49	2.00E-33	0.52	1.26E-37
EPS8L1	EPS8-like 1	-3.6	1.56E-26	5.16E-26	-9.1	3.47E-18	0.65	2.28E-64	0.67	3.67E-68
EPS8L2	EPS8-like 2	-2.2	4.87E-10	7.22E-10	-3.5	6.48E-10	0.56	8.60E-45	0.57	3.98E-46
EVPL	envoplakin	-3.2	7.18E-22	2.02E-21	-5.0	1.03E-17	0.68	2.85E-71	0.69	7.09E-76
FAM3D	family with sequence similarity 3, member D	-16.6	1.69E-173	3.21E-172	-8.5	2.69E-15	0.37	9.92E-19	0.37	1.60E-18
FAM46B	family with sequence similarity 46, member B	-2.4	3.39E-12	5.88E-12	-3.9	1.10E-15	0.68	1.48E-71	0.67	1.41E-68
FAM63A	family with sequence similarity 63, member A	-2.8	7.24E-17	1.63E-16	-2.5	8.92E-14	0.35	1.01E-16	0.39	6.69E-20
FLG	filaggrin	-2.8	3.70E-17	8.45E-17	-5.8	1.50E-07	0.33	4.25E-15	0.35	3.71E-16
FUT3	fucosyltransferase 3 (galactoside 3(4)-L-fucosyltransferase, Lewis blood g	-4.0	1.50E-32	5.80E-32	-5.5	9.92E-19	0.55	1.17E-42	0.58	1.46E-48
GCOM1	GRINL1A complex locus 1	-6.5	8.37E-65	5.80E-64	-4.2	4.81E-13	0.53	1.58E-38	0.56	9.76E-45
GDPD3	glycerophosphodiester phosphodiesterase domain containing 3	-4.9	2.14E-44	1.09E-43	-5.0	2.95E-12	0.63	1.81E-59	0.64	9.48E-61
GGT6	gamma-glutamyltransferase 6	-3.7	1.69E-28	5.91E-28	-2.7	1.33E-07	0.37	3.52E-18	0.36	9.49E-18
GPR110	adhesion G protein-coupled receptor F1	-5.9	8.43E-57	5.17E-56	-2.4	4.26E-05	0.39	1.10E-20	0.42	1.45E-23
GRHL3	grainyhead-like transcription factor 3	-2.7	3.61E-16	7.89E-16	-2.5	7.14E-09	0.55	4.43E-43	0.54	1.63E-41
IL1RN	interleukin 1 receptor antagonist	-4.0	2.02E-32	7.76E-32	-3.5	1.87E-14	0.70	3.04E-77	0.72	1.54E-85
KLK11	kallikrein-related peptidase 11	-2.7	1.21E-15	2.56E-15	-3.5	2.07E-13	0.54	3.73E-40	0.55	8.35E-43
KLK12	kallikrein-related peptidase 12	-3.7	3.12E-28	1.08E-27	-5.3	4.63E-15	0.52	1.48E-37	0.52	5.91E-38
KLK13	kallikrein-related peptidase 13	-5.5	6.10E-52	3.51E-51	-2.4	4.28E-12	0.54	2.33E-41	0.57	6.33E-46
KRT13	keratin 13, type I	-15.6	9.61E-165	1.72E-163	-4.6	5.87E-13	0.33	6.41E-15	0.33	6.74E-15

KRT33B	keratin 33B, type I	-25.5	7.96E-240	2.39E-238	-7.0	6.51E-10	0.33	9.74E-15	0.34	1.67E-15
KRT78	keratin 78, type II	-18.9	3.30E-194	7.41E-193	-19.1	1.81E-25	0.67	5.36E-68	0.67	2.15E-68
LOC643008		-6.1	4.16E-59	2.66E-58	-9.9	4.07E-17	0.54	2.29E-40	0.53	1.28E-39
MAL	mal, T-cell differentiation protein	-30.9	1.60E-281	6.10E-280	-9.5	2.46E-20	0.39	2.44E-20	0.39	9.04E-21
MALL	mal, T-cell differentiation protein-like	-2.5	4.77E-13	8.72E-13	-3.1	3.77E-11	0.61	5.75E-54	0.63	4.58E-58
MAPKAPK3	mitogen-activated protein kinase-activated protein kinase 3	-2.2	5.42E-10	8.01E-10	-2.6	1.27E-09	0.30	2.40E-12	0.31	2.12E-13
MGLL	monoglyceride lipase	-3.9	1.50E-30	5.48E-30	-5.4	6.51E-21	0.37	6.15E-18	0.37	1.96E-18
MPP7	membrane protein, palmitoylated 7 (MAGUK p55 subfamily member 7)	-2.8	2.75E-17	6.34E-17	-2.3	3.58E-09	0.44	3.05E-26	0.46	8.74E-29
MUC15	mucin 15, cell surface associated	-4.7	1.27E-41	6.03E-41	-3.5	6.35E-10	0.39	3.79E-20	0.38	5.54E-19
NMU	neuromedin U	-2.1	5.61E-09	7.72E-09	-2.8	1.85E-08	0.37	1.34E-18	0.35	2.44E-16
PADI1	peptidyl arginine deiminase, type I	-14.2	6.14E-151	9.89E-150	-7.4	2.09E-09	0.39	1.88E-20	0.40	6.31E-21
PALMD	palmdelphin	-2.3	4.49E-11	7.20E-11	-3.1	8.87E-07	0.41	7.63E-23	0.39	3.94E-20
PCDH1	protocadherin 1	-3.0	4.22E-19	1.06E-18	-2.2	1.40E-08	0.39	4.62E-20	0.44	1.42E-25
PDCD4	programmed cell death 4 (neoplastic transformation inhibitor)	-2.5	8.96E-14	1.71E-13	-2.3	7.33E-06	0.37	5.01E-18	0.35	4.30E-16
PINK1	PTEN induced putative kinase 1	-2.0	4.71E-08	6.00E-08	-2.0	4.29E-12	0.47	1.62E-29	0.47	8.98E-30
PITX1	paired-like homeodomain 1	-2.8	3.23E-17	7.40E-17	-7.3	2.55E-21	0.44	8.29E-26	0.46	4.63E-28
PLEKHN1	pleckstrin homology domain containing, family N member 1	-2.3	4.39E-11	7.03E-11	-8.1	2.96E-12	0.61	6.90E-55	0.62	5.22E-57
PPL	periplakin	-5.2	1.28E-47	6.87E-47	-4.1	2.89E-20	0.54	1.50E-40	0.57	6.85E-46
PRDM1	PR domain containing 1, with ZNF domain	-2.0	2.51E-08	3.29E-08	-2.7	2.73E-08	0.44	1.28E-25	0.47	3.49E-30
PRSS27	protease, serine 27	-7.5	2.92E-77	2.39E-76	-9.7	4.05E-12	0.68	4.99E-73	0.70	3.00E-77
PRSS3	protease, serine, 3	-3.6	1.63E-26	5.38E-26	-4.2	2.62E-09	0.35	2.81E-16	0.35	5.06E-16
PTK6	protein tyrosine kinase 6	-2.6	3.14E-14	6.17E-14	-3.9	1.12E-18	0.57	5.30E-47	0.60	6.96E-53
RAB25	RAB25, member RAS oncogene family	-2.2	1.63E-10	2.50E-10	-3.3	1.23E-11	0.65	4.26E-64	0.65	1.68E-64
RAET1E	retinoic acid early transcript 1E	-3.9	5.87E-31	2.18E-30	-4.6	8.67E-13	0.68	8.08E-73	0.69	4.35E-75
RDH12	retinol dehydrogenase 12 (all-trans/9-cis/11-cis)	-2.1	3.16E-09	4.44E-09	-2.9	0.0007311	0.53	1.38E-39	0.52	4.23E-38
RHBG	Rh family, B glycoprotein (gene/pseudogene)	-4.4	4.82E-35	1.99E-34	-3.6	7.83E-09	0.43	1.98E-25	0.43	1.83E-24
RHCG	Rh family, C glycoprotein	-7.6	1.93E-78	1.59E-77	-6.4	2.36E-19	0.60	7.02E-52	0.61	5.27E-54
RMND5B	required for meiotic nuclear division 5 homolog B	-2.5	1.83E-13	3.44E-13	-2.9	1.25E-16	0.39	3.81E-20	0.40	1.30E-21
S100A14	S100 calcium binding protein A14	-2.7	1.41E-15	2.98E-15	-5.2	4.66E-27	0.66	7.88E-66	0.65	6.05E-64
SBSN	suprabasin	-2.6	1.51E-14	3.02E-14	-2.7	1.69E-06	0.71	1.35E-80	0.70	6.70E-78
SCEL	sciellin	-7.3	2.42E-75	1.92E-74	-4.6	1.72E-10	0.63	4.24E-60	0.65	1.83E-63
SCNN1B	sodium channel, non voltage gated 1 beta subunit	-5.5	5.66E-52	3.27E-51	-6.2	5.55E-22	0.40	3.24E-21	0.40	3.46E-21
SERPINB13	serpin peptidase inhibitor, clade B (ovalbumin), member 13	-3.1	3.27E-20	8.55E-20	-2.2	9.65E-07	0.52	5.48E-38	0.54	1.89E-41
SERPINB2	serpin peptidase inhibitor, clade B (ovalbumin), member 2	-2.9	1.08E-18	2.67E-18	-2.2	8.07E-06	0.52	3.64E-37	0.54	5.02E-40
SLC5A1	solute carrier family 5 (sodium/glucose cotransporter), member 1	-2.5	7.95E-14	1.52E-13	-2.2	0.0002827	0.53	1.18E-38	0.54	4.57E-40
SLPI	secretory leukocyte peptidase inhibitor	-3.1	4.44E-21	1.21E-20	-2.1	4.71E-07	0.71	8.88E-82	0.73	1.38E-86
SLURP1	secreted LY6/PLAUR domain containing 1	-6.4	4.75E-63	3.22E-62	-3.3	4.87E-09	0.63	6.36E-60	0.62	1.81E-57
SPINK5	serine peptidase inhibitor, Kazal type 5	-8.8	4.08E-93	3.94E-92	-2.6	9.94E-09	0.58	1.62E-47	0.58	2.89E-48
SPINK7	serine peptidase inhibitor, Kazal type 7 (putative)	-4.9	3.01E-44	1.52E-43	-5.0	1.20E-09	0.60	8.97E-53	0.61	1.58E-54
SPRR2B	small proline-rich protein 2B	-2.4	1.75E-12	3.09E-12	-2.2	0.00169	0.67	9.73E-69	0.67	4.38E-68
SPRR2C	small proline-rich protein 2C (pseudogene)	-3.4	4.88E-24	1.48E-23	-4.3	6.18E-05	0.65	4.82E-64	0.67	7.41E-68
SPRR3	small proline-rich protein 3	-11.2	8.09E-120	1.01E-118	-2.4	8.44E-09	0.43	2.64E-25	0.45	7.31E-27
SULT2B1	sulfotransferase family, cytosolic, 2B, member 1	-2.9	4.53E-18	1.09E-17	-5.3	8.28E-16	0.74	2.89E-90	0.74	2.43E-91
TEX101	testis expressed 101	-3.0	3.62E-18	8.74E-18	-3.6	1.35E-06	0.45	3.44E-27	0.43	2.12E-24
TGM1	transglutaminase 1	-3.5	6.01E-26	1.95E-25	-5.1	6.71E-14	0.74	6.30E-91	0.75	1.14E-96
TGM3	transglutaminase 3	-13.6	8.66E-146	1.31E-144	-16.0	1.03E-12	0.48	9.73E-32	0.48	3.75E-31
TGM5	transglutaminase 5	-3.1	3.26E-21	8.90E-21	-2.8	8.71E-08	0.61	8.92E-54	0.61	3.69E-55
TM7SF2	transmembrane 7 superfamily member 2	-3.6	1.12E-27	3.80E-27	-2.9	2.40E-08	0.38	1.01E-19	0.38	9.97E-20
TMEM125	transmembrane protein 125	-3.2	9.40E-22	2.64E-21	-3.6	8.45E-15	0.45	7.42E-28	0.45	1.41E-27
TMEM40	transmembrane protein 40	-2.3	5.93E-11	9.42E-11	-4.7	9.88E-14	0.70	2.99E-77	0.71	9.70E-80
TMPRSS11B	transmembrane protease, serine 11B	-35.9	3.34534e-311	0	-9.2	5.90E-17	0.36	3.42E-17	0.35	2.89E-16
TMPRSS11D	transmembrane protease, serine 11D	-2.6	3.80E-14	7.43E-14	-3.5	1.32E-12	0.63	2.38E-58	0.64	9.29E-61
TRIP10	thyroid hormone receptor interactor 10	-2.5	2.08E-13	3.89E-13	-2.7	1.14E-10	0.42	1.87E-23	0.43	2.60E-24
TTC9	tetratricopeptide repeat domain 9	-3.9	2.68E-30	9.73E-30	-3.2	4.20E-09	0.46	2.75E-28	0.47	5.40E-30
USP2	ubiquitin specific peptidase 2	-2.7	5.15E-16	1.11E-15	-2.1	0.002335	0.46	1.86E-28	0.47	2.00E-29
WWC1	WW and C2 domain containing 1	-2.7	2.56E-15	5.32E-15	-2.4	1.08E-08	0.32	6.87E-14	0.36	4.06E-17
ZFP36	ZFP36 ring finger protein	-2.8	1.06E-16	2.38E-16	-2.5	3.64E-07	0.35	2.97E-16	0.37	4.48E-18
ZNF750	zinc finger protein 750	-2.5	8.30E-13	1.50E-12	-2.9	1.45E-12	0.58	3.14E-48	0.60	1.13E-51

Table S4. Description and known functions of putative HNSCC marker genes. Genes not detectable in normal mucosal samples but upregulated in HNSCC from the microarray data are shown along with their descriptions, fold-changes (FC) and associated p-values, and known biological functions. FC and p-values in HNSCC from TCGA RNA-Seq V2 data are also shown. FC is measured relative to the normal samples.

				RNA-Seq V2 (TCGA)		
**Gene**	**Description**	**FC**	**p-value**	**FC**	**p-value**	**Functions**
MMP1	Matrix metallopeptidase 1 (interstitial collagenase)	299.5	3.94E-05	8.0	1.35E-28	Extracellular matrix remodeling
INHBA	Inhibin, beta A (activin family)	29.3	1.41E-03	20.3	3.60E-48	Proliferation / differentiation
FST	Follistatin (binds/regulated by INHBA)	7.1	1.00E-04	6.6	8.02E-25	Proliferation / differentiation
LAMC2	Laminin, gamma 2	15.5	1.51E-05	11.8	1.39E-36	Extracellular matrix, adhesion
CCL3	Chemokine (C-C motif) ligand 3	17.0	1.84E-03	2.1	2.88E-06	Inflammation, migration
PTGS2	Prostaglandin-endoperoxide synthase 2 (COX-2)	30.3	4.33E-02	3.3	1.61E-12	Inflammation, mitogenesis
SULF1	Sulfatase 1	4.7	3.37E-02	4.3	5.37E-17	Mitogenesis, motility
SLC16A1	Solute carrier family 16, member 1	4.2	2.27E-04	2.6	6.72E-09	Acidification, membrane transport
